# Fragment-based design of selective GPCR ligands guided by free energy simulations†

**DOI:** 10.1039/d1cc03202j

**Published:** 2021-11-19

**Authors:** Pierre Matricon, Duc Duy Vo, Zhan-Guo Gao, Jan Kihlberg, Kenneth A. Jacobson, Jens Carlsson

**Affiliations:** a Science for Life Laboratory, Department of Cell and Molecular Biology, Uppsala University, Uppsala SE-751 24, Sweden.; b Molecular Recognition Section, Laboratory of Bioorganic Chemistry, National Institute of Diabetes and Digestive and Kidney Diseases, National Institutes of Health, Bethesda, Maryland 20892, USA.; c Department of Chemistry – BMC, Uppsala University, Uppsala SE-751 23, Sweden

## Abstract

Fragment-based drug discovery relies on successful optimization of weakly binding ligands for affinity and selectivity. Herein, we explored strategies for structure-based evolution of fragments binding to a G protein-coupled receptor. Molecular dynamics simulations combined with rigorous free energy calculations guided synthesis of nanomolar ligands with up to >1000-fold improvements of binding affinity and close to 40-fold subtype selectivity.

Technologies that enable more efficient generation of lead candidates are needed to increase the success rate of drug discovery. The small fraction of chemical space that can be screened experimentally continues to be a major limitation. Even if high-throughput screening (HTS) and DNA-encoded libraries contain millions to billions of compounds, these only explore a small fraction of the 10^60^ possible drug-like molecules and will lack chemical starting points for many targets.^1,2^ Fragment-based drug discovery (FBDD) takes an alternative route to identify leads, which has already led to several clinical candidates.^3^ By first screening compounds that are less than half the size of a drug, fragment libraries can provide better coverage of chemical space.^2^ Another advantage is that fragments are likelier to bind to a protein than drug-like compounds because of their small size and low molecular complexity.^4^ However, the high hit rates from fragment screening comes at a price – the compounds will bind weakly and not be selective for the target.^5^ The second step of FBDD, fragment-to-lead optimization, can be very challenging, in particular if a crystal structure of the protein–fragment complex is not available. The dependence of FBDD on high resolution structures has limited the applicability of the method for important drug targets such as transmembrane receptors.^6^ For these reasons, accurate computational models of fragment binding and methods to guide optimization would be valuable. Several recent studies suggest that relative binding free energies calculated from molecular dynamics (MD) simulations can guide hit-to-lead optimization for important drug targets such as G protein-coupled receptors (GPCRs).^7–9^

In this work, we undertook the challenge to optimize fragments binding to a GPCR with the goal to investigate three central questions in FBDD. Firstly, can atomic resolution GPCR structures guide fragment optimization? Crystal structures of numerous GPCR drug targets have recently been solved,^10^ but as complexes with fragments remain scarce, we used MD simulations to model receptor–fragment interactions. As there are multiple GPCR sub-types recognizing the same ligand, it was essential to achieve both affinity and selectivity. Our second question was if MD simulations can be used to model how binding affinity and selectivity is affected by small changes to a fragment’s chemical structure. We assessed if rigorous free energy methods could predict the affinities of evolved fragments. Finally, we analysed advantages of using FBDD to develop chemical probes. A prospective study was performed by iteratively designing elaborated fragments based on the receptor structures and performing free energy calculations. Compounds were synthesized and tested in pharmacological assays, followed by analysis of the accuracy of the computational predictions.

Recently determined crystal structures of the A_1_ and A_2A_ adenosine receptors (A_1_- and A_2A_ARs) could facilitate development of drugs to treat cancer, CNS, and cardiovascular diseases.^11,12^ Prior to the release of the A_1_AR structure, we discovered a fragment (benzothiazole **1**) binding to this GPCR with an affinity of 11.2 μM (Fig. 1).^9^ Docking of the fragment to the A_1_AR binding site suggested that it was anchored by hydrogen bond interactions with Asn254. The ligand interacted with residues that are conserved in both receptors, but the structures revealed a residue substitution that created a unique hydrophobic subpocket in the A_1_AR close to the amide moiety of the fragment. This pocket is located the extracellular entrance of the binding site and was not accessible in the A_2A_AR because Thr270 is replaced by the bulkier side chain of Met270 (Fig. 1). The fragment only had 13 heavy atoms (HAs), corresponding to a ligand efficiency (LE, binding free energy per heavy atom) of 0.52 kcal mol^−1^ HA^−1^, which would be considered to be an excellent starting point for FBDD.^13^

The pocket identified in the A_1_AR crystal structure guided optimization of the fragment. Compounds **2–10** explored a single growth vector by positioning hydrophobic substituents of varying size and shape in the pocket (Fig. 1). The calculations were performed to predict binding free energies of these compounds relative to **1**. MD simulations were initiated from an A_1_AR crystal structure,^11^ which was equilibrated in the presence of a lipid bilayer and water. In the simulations, predicted ligands were alchemically transformed into **1** in complex with the A_1_AR and in aqueous solution using the free energy perturbation (FEP) technique, which can be used to calculate relative binding affinities based on a thermodynamic cycle.^9^ The FEP calculations were carried out using the program Q,^14^ and an average simulation length of >0.4 μs was used for each compound pair. Detailed simulation protocols are described in the ESI.† FEP predicted that all the designed compounds would show improved A_1_AR affinity, but there was a large variation in the magnitude of the gain of binding free energy (Fig. 1 and Tables S1, S2, ESI†). Subsequently, **2–10** were synthesized, and evaluated in radioligand binding assays at the A_1_AR. Detailed synthesis and assay procedures are available in the ESI.† As predicted by FEP, all compounds had improved affinity compared to **1** (Fig. 1). The three compounds with the smallest substituents (**2–4**) displayed the smallest gains of binding, which agreed with the FEP predictions. However, the computational ranking of the larger substituents (**5–10**) did not correlate well with the experimental data. Except in the case of compound **5**, the free energy gain was overestimated by the simulations. Pivaloylated **5** had the highest affinity (*K*_i_ = 285 nM), corresponding to a 39-fold improvement, and the LE increased from 0.52 to 0.56 kcal mol^−1^ HA^−1^. Compound **9** (*K*_i_ = 619 nM) was slightly weaker than **5**, but also retained a high LE (0.50 kcal mol^−1^ HA^−1^).

Based on the first set of compounds, **5** and **9** were further elaborated. Interestingly, binding data (Table S1, ESI†) showed that these ligands were not more selective for the A_1_AR compared to **1** despite that both positioned substituents in the non-conserved pocket (Fig. 1). MD simulations of the complexes with the A_1_AR and A_2A_AR indicated that this result was due to the small size of these ligands. The ligands were able to adopt slightly different binding modes in the receptors, which reduced clashes with Met270 in the A_2A_AR. In a second step, we explored other growth vectors in the binding site to improve selectivity (Fig. 1), which involved adding substituents at either the 4-, 5-, or 6-position of the benzothiazole moiety. The rationale behind these designs was that improved anchoring of the fragments would rigidify the binding mode and thereby enhance the effect of having substituents in the non-conserved pocket, potentially leading to selectivity. As **5** had the highest affinity, the first series of elaborations focused on optimizing this fragment. The FEP protocol was used to calculate binding free energies relative to **5** in the A_1_AR and A_2A_AR binding sites to predict changes in affinity as well as selectivity. A methyl substituent was first introduced at three different positions (compound **11–13**) of the benzothiazole moiety to probe the possible growth vectors (Fig. 1). The simulation results indicated that the 5-position was a hotspot for increasing A_1_AR affinity. As FEP also indicated that selectivity could be improved, the synthesis (see ESI† for details) was focused on substituents at this position (Fig. 1). The experimental binding data showed that FEP correctly predicted that the 5-position (**13**) led to the largest improvement of A_1_AR affinity among the three methyl substituents (Fig. 1 and Tables S3, S4, ESI†). The large loss of affinity for **11** was not captured by FEP, but the ranking of the three compounds by affinity was correct. Several of the substitutions at the 5-position (**13–18**) resulted in improved A_1_AR selectivity and affinity, which was predicted by FEP in a majority of the cases. The lack of improvement of A_1_AR selectivity for **19**, which had a 4-methoxy substituent, was also in agreement with the calculated free energies. Compound **15**, which had a 5-bromo substituent, showed the highest A_1_AR affinity (40 nM), was 19-fold selective, and had a remarkable LE of 0.60 kcal mol^−1^ HA^−1^.

Compound **9** was optimized in two steps and involved synthesis and experimental evaluation of six additional compounds (see ESI† for details, Table S3, ESI†). As **9** had weaker affinity than **5** at the A_1_AR, a methyl group was added to the cyclopentyl moiety to mimic the *tert*-butyl group of **5**. The resulting compound **20** was only 6-fold selective for the A_1_AR, but its experimental affinity improved to 95 nM (Fig. 1). Additional FEP calculations of relative affinities and selectivity were performed for substituents at the 5-position, which led to synthesis of **21–25**. Compound **22**, which had a 5-bromo substituent, resulted in the highest affinity (*K*_i_ = 10 nM) and a 38-fold selectivity for the A_1_AR (Fig. 2). FEP predicted that **22** would have higher affinity than **20**, but only indicated a slight increase of selectivity (Fig. 1 and Tables S3, S4, ESI†). The overall improvement of **22** was astonishing. Addition of six heavy atoms to compound **1** resulted in >1000-fold increase of affinity, and selectivity was improved from 7- to 38-fold. MD simulation snapshots provided an explanation of the high affinity and selectivity of **22**. The bulky 1-methylcyclopentyl substituent of **22** led to clashes with Met270 in the A_2A_AR, which pushed the compound towards the extracellular loops. In contrast, this substituent fitted very well in the A_1_AR pocket with the 5-bromo substituent buried deeply in the binding site (Fig. 3).

In a final set of simulations, we explored if selectivity could instead be shifted towards the A_2A_AR subtype. In fact, benzothiazoles have generally been described as an A_2A_AR selective scaffold.^15^ Compound **10** showed weak 2-fold A_2A_AR selectivity. To test if selectivity could be further increased for the A_2A_AR, compound **26**, which had a 4-methoxy group on the benzothiazole ring, was synthesized (see ESI† for details). FEP predicted this substituent to shift affinity and selectivity further towards A_2A_AR, which was also confirmed experimentally (106 nM, 13-fold selectivity, Fig. 2 and Tables S3, S4, ESI†).

Functional assays measuring intracellular concentrations of cAMP were performed at the human A_1_AR and A_2A_AR to determine the efficacy of compounds **22** and **26**. The experiments demonstrated that the compounds acted as antagonists of both receptors and the selectivity profiles were the same as in the binding assays (Fig. S1 and S2, ESI†). These results also agreed with the fact that the simulations were performed using an inactive receptor conformation.

To evaluate the overall performance of the FEP calculations in the affinity and selectivity optimization, the correlation between predicted and experimental binding free energies were evaluated (Fig. 4 and Tables S2, S4, ESI†). For the affinity optimization at A_1_AR the relative binding free energies were predicted with a mean unsigned error (MUE) of 1.08 kcal mol^−1^, and there was a strong spearman rank correlation (*ρ* = 0.80) for the 24 compounds with a determined affinity. Predictions of selectivity were evaluated based on the difference between relative binding free energies for the two receptors from FEP and experiment, and in this case the MUE and *ρ* were 0.48 kcal mol^−1^ and 0.85, respectively, for the compounds with determined affinities. Hence, there was a good correlation between FEP and experiment for both affinity and selectivity. Returning to the three questions that motivated this study, we first conclude that computational models of GPCR–fragment complexes successfully guided fragment elaboration. Structure-informed selection of substituents led to a remarkable >1000-fold improvement of affinity and close to 40-fold receptor subtype selectivity. Second, the relative binding free energies calculated with FEP accurately ranked compounds by affinity as well as selectivity, suggesting that MD simulations can be a useful tool to identify which fragments to elaborate and guide selection of substituents. The approach is most suitable for receptors with a well-defined binding site that recognize fragment-sized endogenous ligands (*e.g*. aminergic GPCRs). Applications to other targets (*e.g*. peptide or protein-binding GPCRs) may be more challenging because fragments bind weakly and modelling of binding modes will be difficult. Finally, comparison of **22** to previously developed A_1_AR ligands clearly illustrates the benefits of using FBDD compared to HTS, which initiates optimization from larger lead-like compounds. In fact, analysis of reported A_1_AR ligands of the same size as **22** (<20 HA) showed that this compound is one of the highest affinity ligands of this size ever discovered (Table S5, ESI†). By carefully evolving fragments, atom-by-atom, high affinity leads can be obtained by synthesizing a small series of compounds, and the chances of obtaining leads with favourable physicochemical properties increase.

This project has received funding from the European Research Council (ERC) under the European Union’s Horizon 2020 research and innovation programme (grant agreement: 715052). The work was also supported by the Swedish Research Council (2017–4676) and the Swedish strategic research programme eSSENCE. K. A. J. thanks the National Institute of Diabetes and Digestive and Kidney Diseases (NIDDK) Intramural Research Program (ZIADK31117) for financial support. Computational resources were provided by the Swedish National Infrastructure for Computing (SNIC).

## Supplementary Material

Supplementary material

## Figures and Tables

**Fig. 1:**
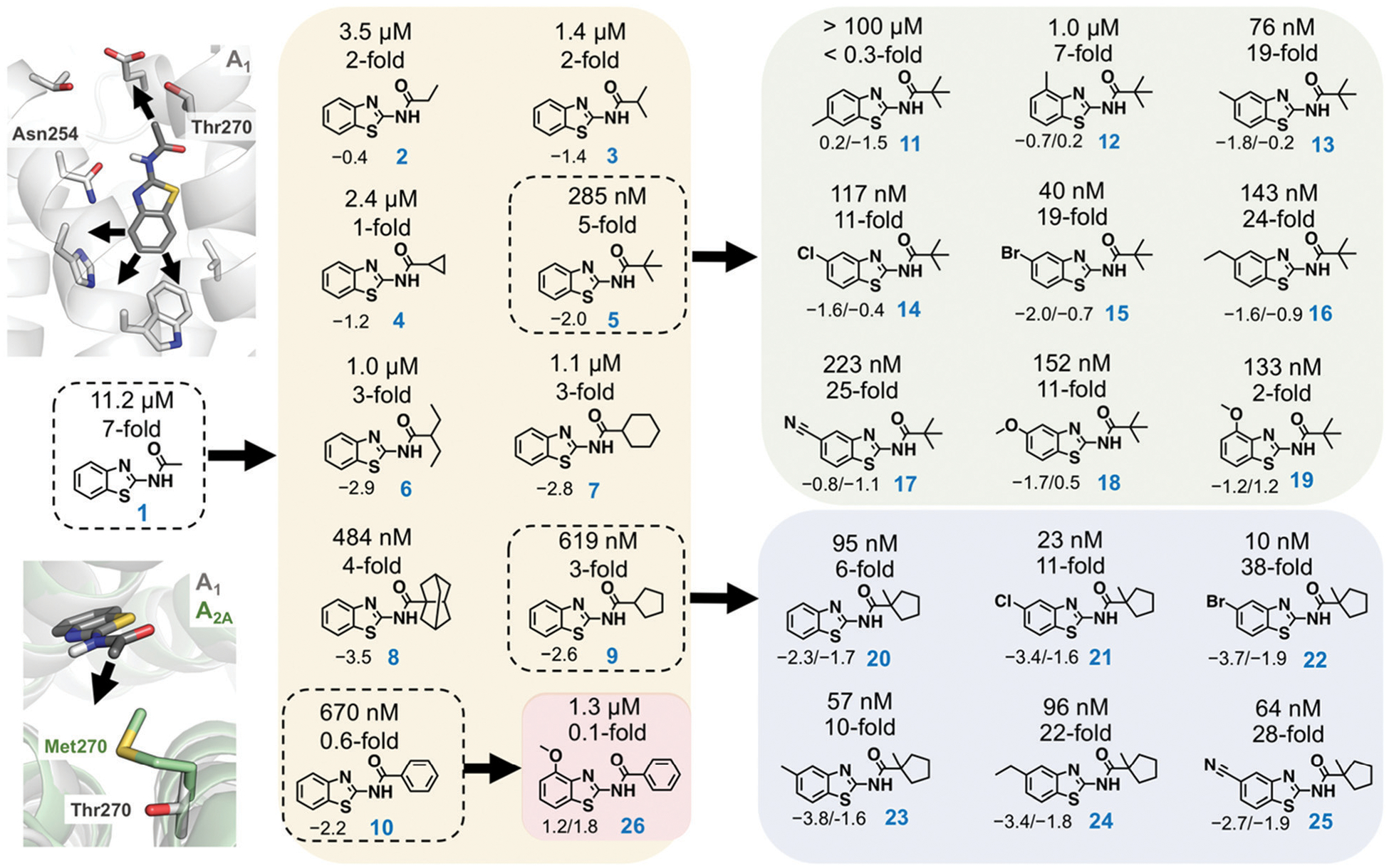
Fragment-based lead design guided by free energy simulations. Growth vectors to evolve compound 1 were identified in the A_1_AR binding site. A first series of compounds explored a unique subpocket of the A_1_AR at the extracellular entrance of the binding site (2–10). Two additional series of compounds resulted in high affinity and subtype selective ligands (11–26). The experimentally determined binding affinities (*K*_i_ values) and selectivity for the A_1_AR are shown above each compound. The compounds used as references for the FEP calculations are marked with black dashed lines. For compounds 2–10, the calculated relative binding free energies (kcal mol^−1^) at the A_1_AR are shown below each compound and negative values indicate improved affinity. For compounds 11–26, the relative binding free energies at the A_1_AR and the difference in relative binding free energy between A_1_AR and A_2A_AR (negative values indicate improved A_1_AR selectivity) are shown below each compound (affinity/selectivity, kcal mol^−1^). The experimental and calculated binding data are also summarized in Tables S1–S4 (ESI†).

**Fig. 2:**
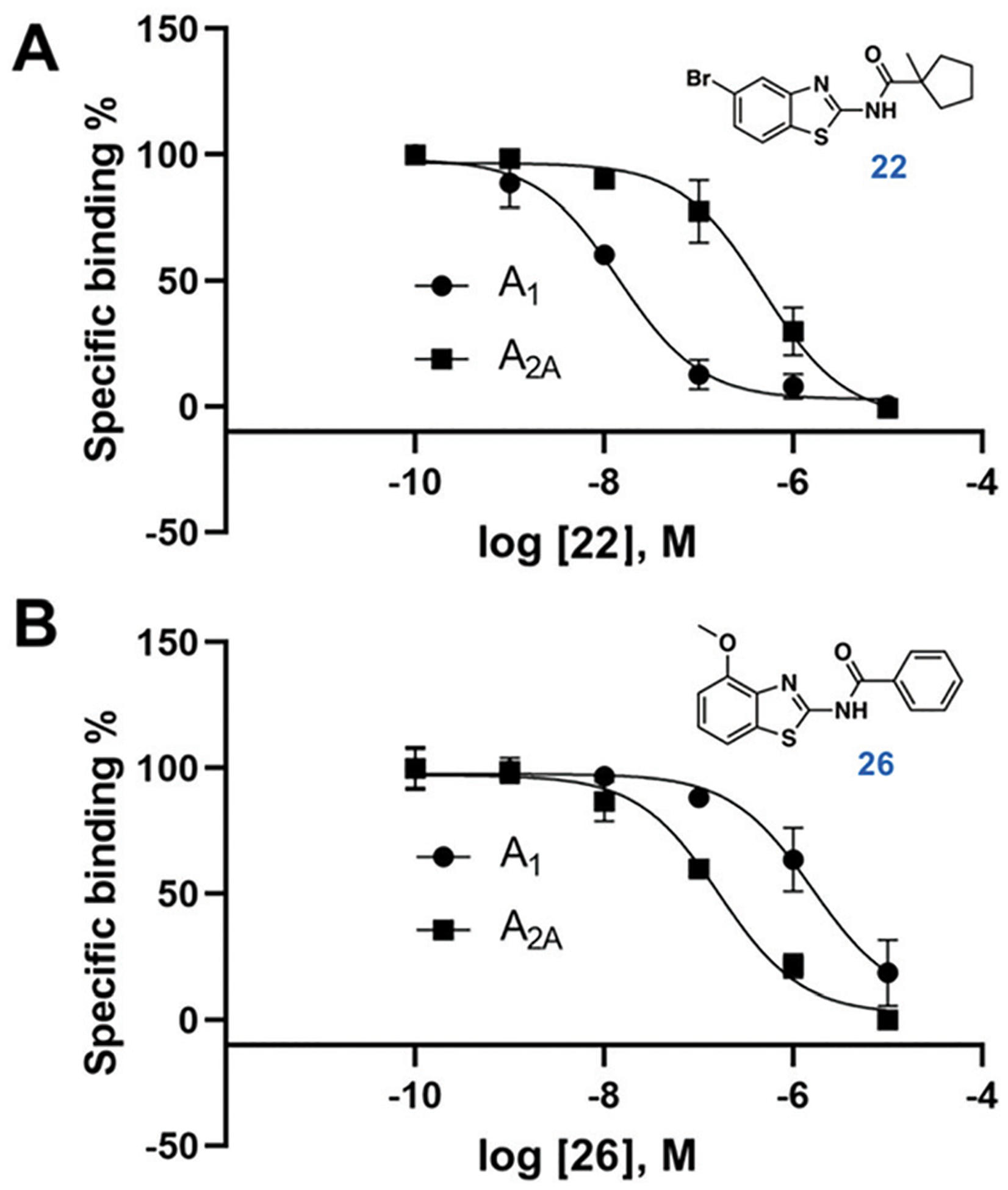
Dose–response curves from radioligand binding assays for compounds 22 and 26 (*n* = 2–4).

**Fig. 3:**
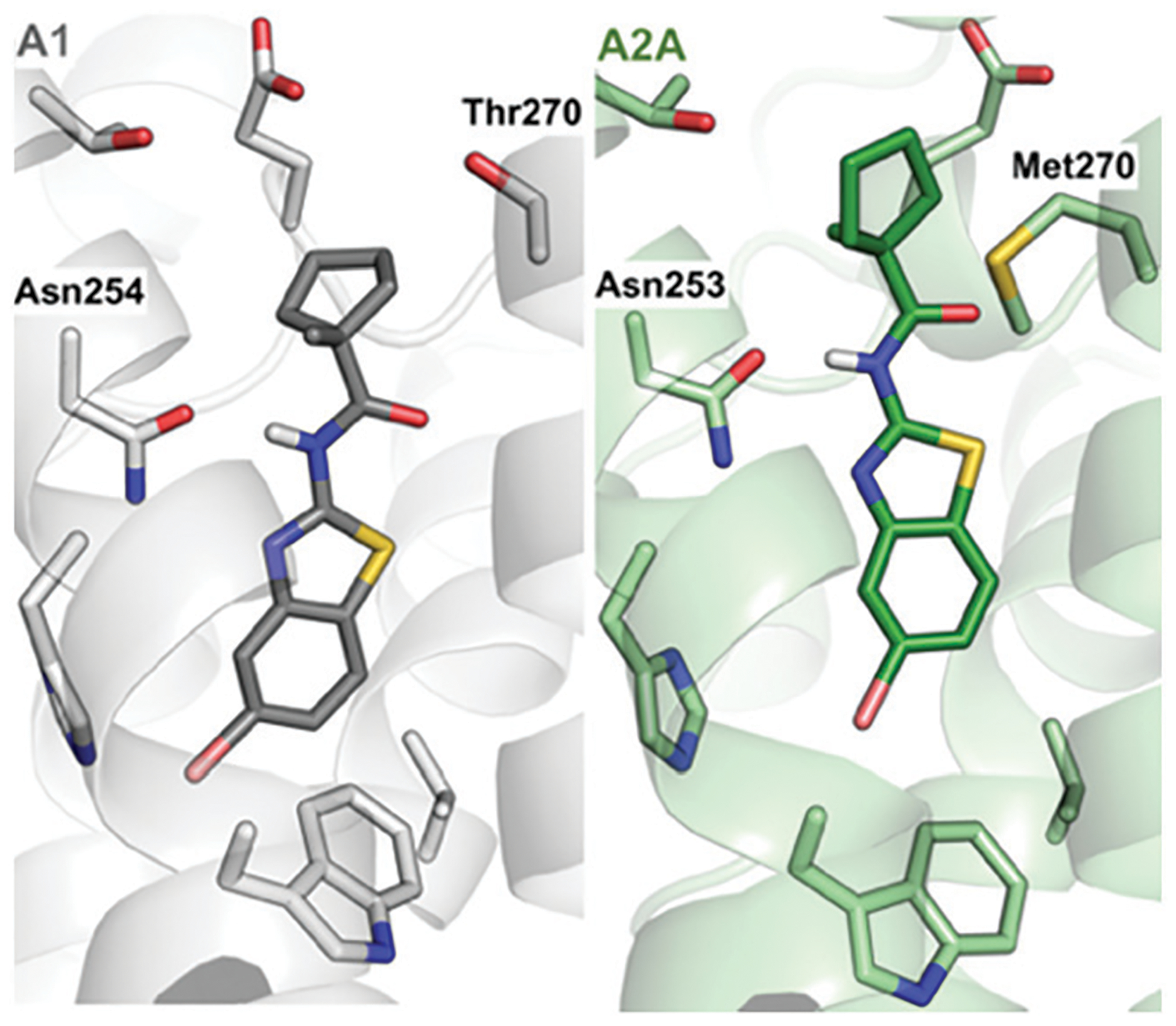
Predicted structures of compound 22 bound to the A_1_- (grey) and A_2A_AR (green).

**Fig. 4:**
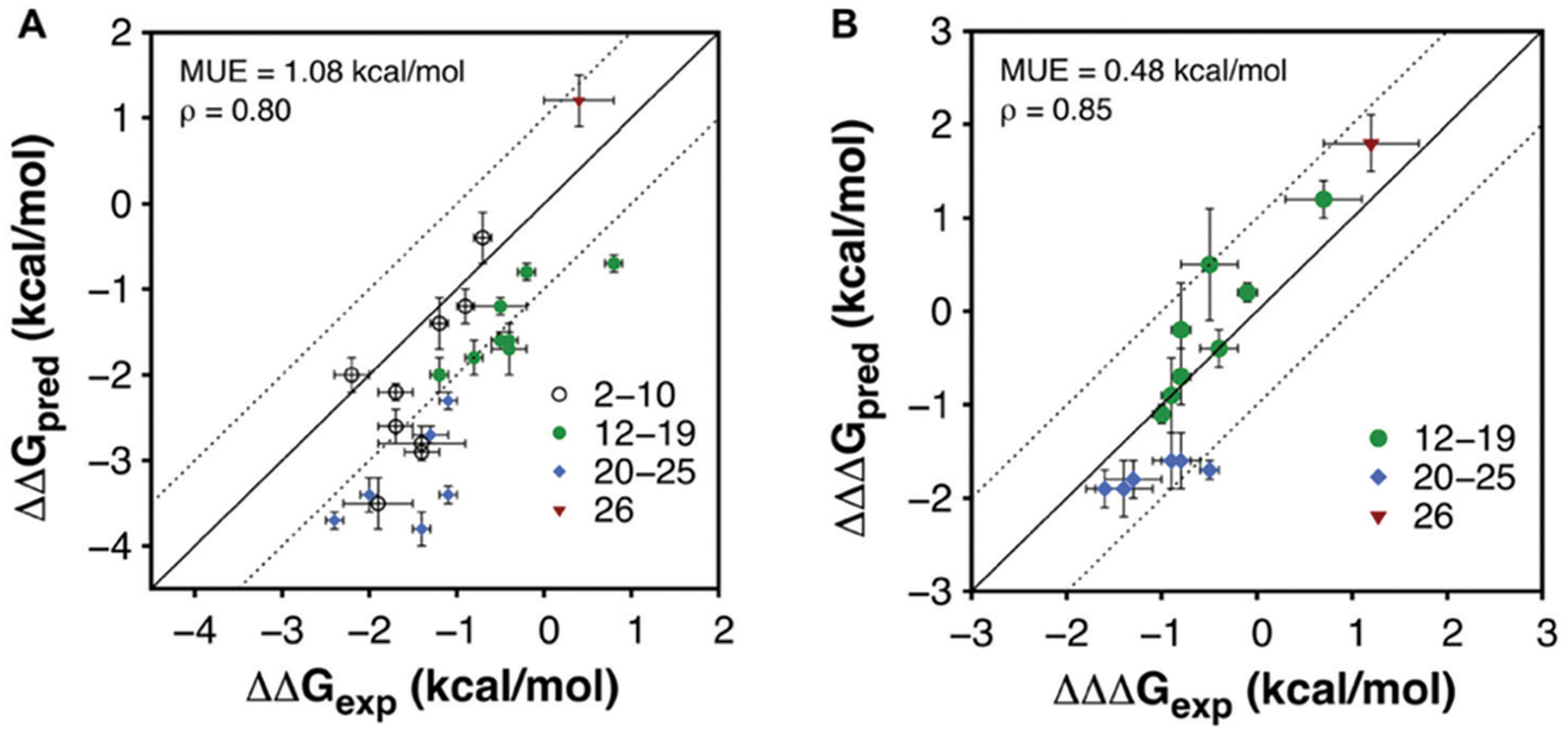
Correlation between experimental and calculated free energies (A) for affinity and (B) selectivity at the A_1_AR.
