# Supplementary material for: Fragment-based design of selective GPCR ligands guided by free energy simulations
Source: Chem Commun (Camb). Author manuscript; Available in PMC 2022 Nov 19. (PMC8603191; doi:10.1039/d1cc03202j)
Supplement: Supplementary material [file NIHMS1758126-supplement-Supplementary_material.pdf]

Electronic Supplementary Material (ESI) for Chemical Communications.
This journal is © The Royal Society of Chemistry 2021

Supplementary Information

Fragment-based design of selective GPCR ligands guided by free energy simulations

Pierre Matricon[a], Duc Duy Vo[a], Zhan-Guo Gao[b], Jan Kihlberg[c], Kenneth A. Jacobson[b], and Jens Carlsson[a,*]

[a]Science for Life Laboratory, Department of Cell and Molecular Biology, Uppsala University, SE-751 24 Uppsala, Sweden.

[b]Molecular Recognition Section, Laboratory of Bioorganic Chemistry, National Institute of Diabetes and Digestive and Kidney Diseases, National Institutes of Health, Bethesda, Maryland 20892, USA.

[c]Department of Chemistry - BMC, Uppsala University, SE-751 23 Uppsala, Sweden.

*E-mail: jens.carlsson@icm.uu.se

Table of Contents

Supplementary Methods and Results

Computational chemistry S3

Chemistry S5

Biological assays S13

Supplementary Figures

Functional assay for compound **22** S14

Functional assay compound **26** S15

Supplementary Tables

Experimental binding affinities of compounds **1**-**10** S16

Relative binding free energies for compounds **2**-**10** S17

Experimental binding affinities of compounds **11-26** S18

Relative binding free energies for compounds **11**-**26** S19

High affinity A_1AR ligands with < 20 heavy atoms S20

NMR Spectra

Compounds synthesized at Enamine (**2-6** and **9-10**) S21

Compounds synthesized in-house (**7-8** and **11-26**) S28

Supplementary References S46

Supplementary Methods and Results

Computational Chemistry

Molecular dynamics simulations and free energy calculations. Simulations of compounds bound to the A_1AR and $A_{2A}AR$ were based on crystal structures of the receptors in complex with PSB36 (PDB code: 5N2S)[1] and tozadenant (PDB code: 5OLO)[2], respectively. Engineered mutations were reverted and non-protein atoms were removed prior to initiating the simulations. Each receptor was then placed in a pre-equilibrated 1-palmitoyl-2-oleoyl-phosphatidylcholine (POPC) lipid bilayer using the GPCR-ModSim protocol[3]. The resulting systems were equilibrated at 310 K and atmospheric pressure for 40 ns using GROMACS[4]. During this step, protein heavy atoms were held rigid with tight positional restraints whereas membrane and water molecules were equilibrated. The OPLS 2005 all atom force field[5], Berger lipids[6] and the TIP3P water model[7] were used to carry out these simulations. For each receptor-ligand complex, the studied compound was added and clashing waters were removed. The ligand binding modes in the A_1AR binding site were based on a molecular docking pose of compound **1**[8] and the core scaffold of the compound was superimposed with the coordinates of tozadenant for the $A_{2A}AR$. MD simulations were performed with the software Q[9] using its implementation of a newer version of the same protein force field[10] and the same lipid parameters and water model as in the equilibration of the system. For each compound, OPLSAA_2005 force field parameters were obtained from the hetgrp_ffgen program (Schrödinger, LLC, New York, NY, 2017). In these simulations, spherical boundary conditions were used with a 21 Å radius sphere centered on the ligand. All atoms outside the sphere were excluded from non-bonded interactions and ionizable residues at the sphere edge were set to their neutral form. In addition, atoms within 3 Å of the edge of the spherical system were restrained to their initial coordinates. The surface-constrained all atom solvent (SCAAS) model[11] was applied to water molecules at the sphere surface using radial and polarization restraints. Solvent bonds and angles were constrained with the SHAKE algorithm[12] and except for ligand atoms, a cutoff of 10 Å was used for non-bonded

interactions. Long-range electrostatic interactions were then treated with the local reaction field approximation[13]. Non-bonded pair lists were updated every 25 steps using a time step of 1 fs in all simulations. Ionizable residues in the binding site were protonated according to the most probable in aqueous solution at pH 7. For the A_1AR, His78$^{3.23}$, His264^{ECL3} and His278$^{7.42}$ were protonated on the δ position and His251$^{6.52}$ on the ε position. For the $A_{2A}AR$, His264^{ECL3} and His278$^{7.42}$ were protonated on the δ position whereas His75$^{3.23}$, His155^{ECL2} and His250$^{6.52}$ were protonated on the ε position. The alchemical transformations of the compounds were divided into four steps: (i) Transformation of the partial charges, (ii,iii) annihilation of atoms by first introducing a soft-core potential and then removing the resulting Lennard-Jones term for these atoms,[14] and (iv) remaining Lennard-Jones parameters and bonded terms were changed. These calculations were divided into 11, 11, 21 and 41 steps by using a mapping potential (*U*) that describes each transformation as a linear combination of the potential energy functions of the start (A) and end (B) states:

$$U = (1 - \lambda)U_A + \lambda U_B \tag{1}$$

where λ is varied from zero to one. The free energy difference between states was then obtained using the Zwanzig equation.[15] At each window, the receptor-ligand complex was equilibrated for 750 ps with harmonic positional restraints on solute heavy atoms, which were gradually released while the system was heated. Equilibration was followed by 500 ps production runs and energies were collected every 50 fs. The ligands were also prepared for simulations in aqueous solution using a water droplet of the same size. In these simulations, a weak harmonic restraint was applied to a central ligand atom to prevent it from approaching the sphere edge and the systems were equilibrated for 350 ps, which was followed by 100 ps productions. Each calculation was performed using three independent replicates and relative binding free energies were obtained based on a thermodynamic cycle, as described in a previous work.[8] Each free energy difference was calculated using a bootstrapping approach by randomly selecting one of the three

independent replicates at each step of the transformation (1000 rounds). Free energy values represent means ± SD of the results obtained from the 1000 rounds of bootstrapping.

Chemistry

General Synthetic Procedures. All reagents were purchased from Fluorochem, Sigma-Aldrich, Enamine and Chemtronica. For solvents, DCM, methanol, DMF, and acetonitrile (99.9%) were purchased from VWR International AB, whereas THF was purchased from Sigma-Aldrich. Reagents and solvents were used as such without further purification. All reactions involving air or moisture-sensitive reagents or intermediates were performed under a nitrogen atmosphere. LC-MS was used for monitoring reactions and assessing purity using an Agilent 1100 series HPLC having a C18 Atlantis T3 column (3.0 × 50 mm, 5 μm). Acetonitrile–water (flow rate 0.75 mL/min over 6 min) was used as mobile phase. A Waters micromass Z_Q (model code: MM1) mass spectrometer with electrospray ionization was used for detection of molecular ions. Silica gel 60 F_{254} TLC plates from Merck were also used for monitoring reactions and particularly during purification of compounds. Visualization of the developed TLC was performed using UV light (254 nm) and staining with ninhydrin or anisaldehyde. After workup, organic phases were dried over Na_2SO_4/$MgSO_4$ and filtered before being concentrated under reduced pressure. Silica gel (Matrex, 60 Å, 35–70 μm, Grace Amicon) was used for purification of intermediate compounds with flash column chromatography. Preparative reversed-phase HPLC was performed on a Kromasil C8 column (250 × 21.2 mm, 5 μm) on a Gilson HPLC equipped with Gilson 322 pump, UV/Visible-156 detector and 202 collector using acetonitrile-water gradients as eluents with a flow rate of 15 mL/min and detection at 210 or 254 nm. Unless otherwise stated, all the tested compounds were purified by HPLC. ^{1}H and ^{13}C NMR spectra for synthesized compounds were recorded at 298 K on an Agilent Technologies 400 MR spectrometer at 400 MHz or 100 MHz, or on Bruker Avance Neo spectrometers at 500/600 MHz or 125/150 MHz. Chemical shifts are reported in parts per

million (ppm, δ) referenced to the residual ^1H resonance of the solvent ($(CD_3)_2CO$, δ 2.05; $CDCl_3$, δ 7.26; CD_3OD δ 3.31; DMSO-d_6 δ 2.50). Splitting patterns are designated as follows: s (singlet), d (doublet), t (triplet), m (multiplet) and br (broad). Coupling constants (J values) are listed in hertz (Hz). The purity of the tested compounds is >95% as determined by high resolution ^1H NMR spectroscopy (400-600 MHz) and LCMS.

Scheme S1. Synthesis of compounds

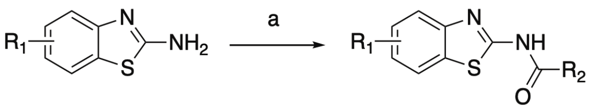

Reagents and conditions: a) RCOCl, pyridine, 0 °C - rt, 1 h, 30-80 % (hplc).

General procedure for amide coupling with acid chlorides, using compound 26 as an example

Benzoyl chloride (29 µL, 1 equiv.) was added to a mixture of 4-methoxy-1,3-benzothiazol-2-amine (45 mg, 0.25 mmol, 1 equiv.) in dry pyridine (1 mL) at 0 °C. The mixture was stirred at rt for 1 h, after which LCMS showed full conversion. The mixture was then diluted with DMSO and filtered. Purification by hplc using 5-100% ACN in H_2O afforded the desired product as white solid. Yield: 44 mg (62%).

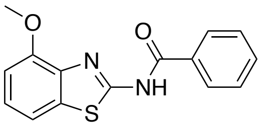

Compound 26. N-(4-methoxybenzo[d]thiazol-2-yl)benzamide.

LCMS (ESI+): calculated for $C_{15}H_{13}N_2O_2S$ $(M+H)^+$: 285.1; found 285.1.

^1H NMR (400 MHz, DMSO-d_6) δ 13.01 (br s, 1H), 8.19-8.12 (m, 2H), 7.70-7.52 (m, 4H), 7.29 (t, J = 8.0 Hz, 1H), 7.04-7.00 (m, 1H), 3.94 (s, 3H).

^{13}C NMR (100 MHz, DMSO-d_6) δ 132.8, 128.6, 128.2, 124.6, 113.3, 107.4, 55.6.

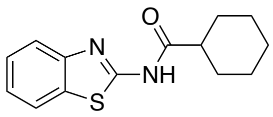

Compound 7. N-(benzo[d]thiazol-2-yl)cyclohexanecarboxamide.

Procedure for compound **26** was applied at 0.2 mmol scale. Yield: 36 mg (69%).

LCMS (ESI+): calculated for $C_{14}H_{17}N_2OS$ $(M+H)^+$: 261.1; found 261.2.

^1H NMR (500 MHz, $CDCl_3$) δ 10.85 (br s, 1H), 8.02-7.26 (m, 4H), 2.58-1.09 (m, 11H).

^{13}C NMR (125 MHz, $CDCl_3$) δ 175.0, 160.2, 146.3, 131.2, 126.6, 124.3, 121.7, 119.9, 44.9, 29.1, 25.4, 25.1.

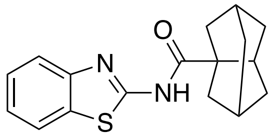

Compound 8. N-(benzo[d]thiazol-2-yl)noradamantanecarboxamide.

Procedure for compound **26** was applied at 0.125 mmol scale. Yield: 10 mg (27%).

LCMS (ESI+): calculated for $C_{18}H_{19}N_2OS$ $(M+H)^+$: 299.1; found 299.2.

^1H NMR (500 MHz, $CDCl_3$) δ 9.56 (br s, 1H), 7.83 (d, *J* = 7.9 Hz, 1H), 7.76 (d, *J* = 8.1 Hz, 1H), 7.46 (td, *J* = 8.0, 0.8 Hz, 1H), 7.34 (t, *J* = 7.8 Hz, 1H), 2.85 (t, *J* = 6.8 Hz, 1H), 2.42 (s, 2H), 2.15-1.63 (m, 10H).

^{13}C NMR (125 MHz, $CDCl_3$) δ 175.8, 158.9, 146.5, 131.4, 126.6, 124.2, 121.6, 120.1, 55.5, 47.2, 43.9, 43.7, 37.6, 34.4.

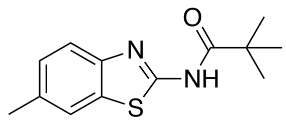

Compound 11. N-(6-methylbenzo[d]thiazol-2-yl)pivalamide.

Procedure for compound **26** was applied at 0.2 mmol scale. Yield: 20 mg (40%).

LCMS (ESI+): calculated for $C_{13}H_{17}N_2OS$ $(M+H)^+$: 249.1; found 249.2.

^1H NMR (500 MHz, $CDCl_3$) δ 7.67 (d, *J* = 8.3 Hz, 1H), 7.62 (s, 1H), 7.31 (d, *J* = 8.3 Hz, 1H), 2.49 (s, 3H), 1.38 (s, 9H).

^{13}C NMR (125 MHz, $CDCl_3$) δ 177.5, 160.1, 135.2, 128.7, 121.5, 118.4, 39.7, 26.9, 21.5.

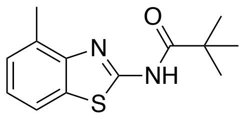

Compound 12. N-(4-methylbenzo[d]thiazol-2-yl)pivalamide.

Procedure for compound **26** was applied. Yield: 40 mg (64%).

LCMS (ESI+): calculated for $C_{13}H_{17}N_2OS$ $(M+H)^+$: 249.1; found 249.2.

^{1}H NMR (400 MHz, $CDCl_3$) δ 9.19 (br s, 1H), 7.65 (d, *J* = 7.4 Hz, 1H), 7.26-7.19 (m, 2H), 2.64 (s, 3H), 1.37 (s, 9H).

^{13}C NMR (100 MHz, $CDCl_3$) δ 176.6, 157.3, 146.9, 131.7, 130.4, 126.9, 123.9, 118.8, 39.4, 27.2, 18.1.

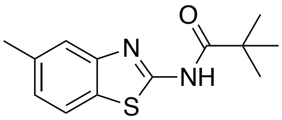

Compound 13. N-(5-methylbenzo[d]thiazol-2-yl)pivalamide.

Procedure for compound **26** was applied at 0.125 mmol scale. Yield: 13 mg (42%).

LCMS (ESI+): calculated for $C_{13}H_{17}N_2OS$ $(M+H)^+$: 249.1; found 249.2.

^{1}H NMR (500 MHz, $CDCl_3$) δ 7.69 (d, *J* = 8.1 Hz, 1H), 7.55 (s, 1H), 7.15 (d, *J* = 8.1 Hz, 1H), 2.48 (s, 3H), 1.36 (s, 9H).

^{13}C NMR (125 MHz, $CDCl_3$) δ 176.6, 158.6, 147.7, 136.5, 128.8, 125.6, 121.0, 120.6, 39.4, 27.1, 21.5.

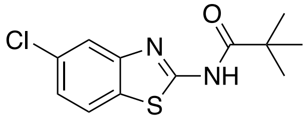

Compound 14. N-(5-chlorobenzo[d]thiazol-2-yl)pivalamide.

Procedure for compound **26** was applied at 0.1 mmol scale. Yield: 14 mg (52%).

LCMS (ESI+): calculated for $C_{12}H_{14}ClN_2OS$ $(M+H)^+$: 269.1; found 269.2.

^{1}H NMR (600 MHz, $CDCl_3$) δ 7.76-7.71 (m, 2H), 7.29 (d, *J* = 8.8 Hz, 1H), 1.36 (s, 3H).

^{13}C NMR (150 MHz, $CDCl_3$) δ 176.8, 159.8, 148.6, 132.4, 130.2, 124.5, 122.2, 120.5, 39.4, 27.1.

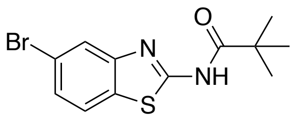

Compound 15. N-(5-bromobenzo[d]thiazol-2-yl)pivalamide.

Procedure for compound **26** was applied at 0.1 mmol scale. Yield: 14 mg (45%).

LCMS (ESI+): calculated for $C_{12}H_{14}BrN_2OS$ $(M+H)^+$: 313.0; found 313.1.

^{1}H NMR (600 MHz, $CDCl_3$) δ 7.89 (s, 1H), 7.67 (d, *J* = 8.4 Hz, 1H), 7.43 (d, *J* = 8.4 Hz, 1H), 1.36 (s, 3H).

^{13}C NMR (150 MHz, $CDCl_3$) δ 176.8, 159.7, 148.7, 130.6, 127.2, 123.4, 122.6, 120.0, 39.4, 27.1.

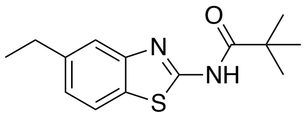

Compound 16. N-(5-ethylbenzo[d]thiazol-2-yl)pivalamide.

Procedure for compound **26** was applied at 0.125 mmol scale. Yield: 14 mg (43%).

LCMS (ESI+): calculated for $C_{14}H_{19}N_2OS$ $(M+H)^+$: 263.1; found 263.2.

^{1}H NMR (500 MHz, $CDCl_3$) δ 7.71 (d, *J* = 8.1 Hz, 1H), 7.58 (s, 1H), 7.18 (d, *J* = 8.1 Hz, 1H), 2.77 (q, *J* = 7.6 Hz, 2H), 1.35 (s, 9H), 1.29 (t, *J* = 7.6 Hz, 3H).

^{13}C NMR (125 MHz, $CDCl_3$) δ 176.6, 158.6, 147.8, 143.0, 129.0, 124.6, 121.1, 119.4, 39.4, 28.9, 27.1, 15.8.

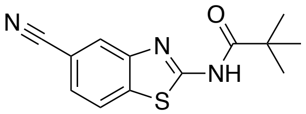

Compound 17. N-(5-cyanobenzo[d]thiazol-2-yl)pivalamide.

Procedure for compound **26** was applied at 0.1 mmol scale. Yield: 11 mg (42%).

LCMS (ESI+): calculated for $C_{13}H_{14}N_3OS$ $(M+H)^+$: 260.1; found 260.2.

^{1}H NMR (600 MHz, $CDCl_3$) δ 8.07 (s, 1H), 7.91 (d, *J* = 8.2 Hz, 1H), 7.54 (d, *J* = 8.2 Hz, 1H), 1.38 (s, 3H).

^{13}C NMR (150 MHz, $CDCl_3$) δ 177.0, 160.1, 147.9, 137.1, 126.4, 124.7, 122.5, 118.9, 110.0, 39.5, 27.1.

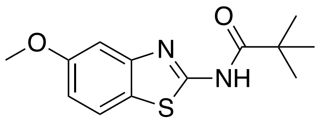

Compound 18. N-(5-methoxybenzo[d]thiazol-2-yl)pivalamide.

Procedure for compound **26** was applied at 0.2 mmol scale. Yield: 20 mg (38%).

LCMS (ESI+): calculated for $C_{13}H_{17}N_2O_2S$ $(M+H)^+$: 265.1; found 265.2.

^{1}H NMR (500 MHz, $CDCl_3$) δ 7.67 (d, *J* = 8.8 Hz, 1H), 7.25 (d, *J* = 2.2 Hz, 1H), 7.04 (d, *J* = 8.8, 2.2 Hz, 1H), 3.89 (s, 3H), 1.39 (s, 9H).

^{13}C NMR (125 MHz, $CDCl_3$) δ 177.7, 162.4, 160.1, 122.3, 115.1, 101.3, 55.9, 39.8, 26.8.

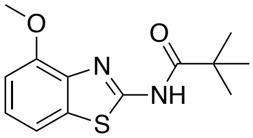

Compound 19. N-(4-methoxybenzo[d]thiazol-2-yl)pivalamide.

Procedure for compound **26** was applied. Yield: 40 mg (60%).

LCMS (ESI+): calculated for $C_{13}H_{17}N_2O_2S$ $(M+H)^+$: 265.1; found 265.2.

^{1}H NMR (400 MHz, $CDCl_3$) δ 9.59 (br s, 1H), 7.39 (d, *J* = 8.0 Hz, 1H), 7.26 (t, *J* = 8.0 Hz, 1H), 6.89 (d, *J* = 8.0 Hz, 1H), 4.01 (s, 3H), 1.34 (s, 9H).

^{13}C NMR (100 MHz, $CDCl_3$) δ 176.6, 157.4, 151.7, 137.1, 133.0, 124.9, 113.4, 106.8, 55.8, 39.4, 27.1.

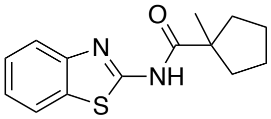

Compound 20. N-(benzo[d]thiazol-2-yl)-1-methylcyclopentane-1-carboxamide.

Procedure for compound **26** was applied at 0.05 mmol scale. Yield: 8 mg (61%).

LCMS (ESI+): calculated for $C_{14}H_{17}N_2OS$ $(M+H)^+$: 261.1; found 261.2.

^{1}H NMR (600 MHz, $CDCl_3$) δ 7.84 (d, *J* = 7.9 Hz, 1H), 7.78 (d, *J* = 8.1 Hz, 1H), 7.50 (t, *J* = 7.6 Hz, 1H), 7.37 (t, *J* = 7.6 Hz, 1H), 2.24-2.16 (m, 2H), 1.81-1.77 (m, 4H), 1.72-1.65 (m, 2H), 1.43 (s, 3H).

^{13}C NMR (150 MHz, $CDCl_3$) δ 177.2, 160.0, 144.5, 130.4, 127.0, 124.7, 121.7, 119.5, 50.5, 37.5, 25.0, 24.9.

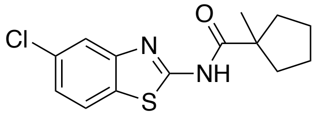

Compound 21. N-(5-chlorobenzo[d]thiazol-2-yl)-1-methylcyclopentane-1-carboxamide.

Procedure for compound **26** was applied at 0.05 mmol scale. Yield: 7 mg (48%).

LCMS (ESI+): calculated for $C_{14}H_{16}ClN_2OS$ $(M+H)^+$: 295.1; found 295.2.

^1H NMR (600 MHz, $CDCl_3$) δ 7.74-7.72 (m, 2H), 7.31 (d, *J* = 8.4 Hz, 1H), 2.22-2.17 (m, 2H), 1.80-1.77 (m, 4H), 1.68-1.64 (m, 2H), 1.41 (s, 3H).

^{13}C NMR (150 MHz, $CDCl_3$) δ 177.0, 160.5, 147.3, 132.7, 129.6, 124.8, 122.4, 120.0, 50.4, 37.6, 25.0, 24.9.

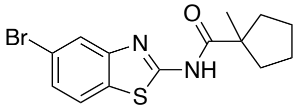

Compound 22. N-(5-bromobenzo[d]thiazol-2-yl)-1-methylcyclopentane-1-carboxamide.

Procedure for compound **26** was applied at 0.05 mmol scale. Yield: 5 mg (30%).

LCMS (ESI+): calculated for $C_{14}H_{16}BrN_2OS$ $(M+H)^+$: 339.0; found 339.1.

^1H NMR (600 MHz, $CDCl_3$) δ 7.90 (s, 1H), 7.68 (d, *J* = 8.4 Hz, 1H), 7.45 (d, *J* = 8.4 Hz, 1H), 2.22-2.17 (m, 2H), 1.80-1.77 (m, 4H), 1.68-1.64 (m, 2H), 1.41 (s, 3H).

^{13}C NMR (150 MHz, $CDCl_3$) δ 177.0, 160.3, 147.5, 130.1, 127.4, 123.0, 122.7, 122.3, 50.4, 37.6, 25.0, 24.9.

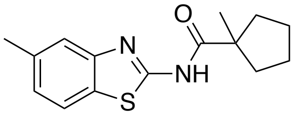

Compound 23. 1-methyl-N-(5-methylbenzo[d]thiazol-2-yl)cyclopentane-1-carboxamide.

Procedure for compound **26** was applied at 0.05 mmol scale. Yield: 10 mg (73%).

LCMS (ESI+): calculated for $C_{15}H_{19}N_2OS$ $(M+H)^+$: 275.1; found 275.2.

^1H NMR (600 MHz, $CDCl_3$) δ 7.70 (d, *J* = 8.1 Hz, 1H), 7.58 (s, 1H), 7.21 (d, *J* = 8.1 Hz, 1H), 2.49 (s, 3H), 2.26-2.16 (m, 2H), 1.80-1.77 (m, 4H), 1.70-1.65 (m, 2H), 1.43 (s, 3H).

^{13}C NMR (150 MHz, $CDCl_3$) δ 177.3, 160.7, 143.6, 137.7, 126.8, 126.4, 121.3, 119.1, 50.6, 37.5, 24.9, 21.5.

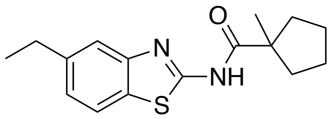

Compound 24. N-(5-ethylbenzo[d]thiazol-2-yl)-1-methylcyclopentane-1-carboxamide.

Procedure for compound **26** was applied at 0.05 mmol scale. Yield: 7 mg (49%).

LCMS (ESI+): calculated for $C_{16}H_{21}N_2OS$ $(M+H)^+$: 289.1; found 289.2.

^1H NMR (600 MHz, $CDCl_3$) δ 7.72 (d, *J* = 8.2 Hz, 1H), 7.60 (s, 1H), 7.23 (d, *J* = 8.2 Hz, 1H), 2.79 (q, *J* = 7.6 Hz, 2H), 2.23-2.17 (m, 2H), 1.80-1.75 (m, 4H), 1.70-1.65 (m, 2H), 1.42 (s, 3H), 1.30 (t, *J* = 7.6 Hz, 3H).

^{13}C NMR (150 MHz, $CDCl_3$) δ 177.2, 160.4, 144.0, 127.3, 125.3, 121.4, 118.1, 50.6, 37.5, 28.9, 24.9, 15.7.

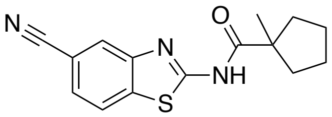

Compound 25. N-(5-cyanobenzo[d]thiazol-2-yl)-1-methylcyclopentane-1-carboxamide.

Procedure for compound **26** was applied at 0.05 mmol scale. Yield: 5 mg (35%).

LCMS (ESI+): calculated for $C_{15}H_{16}N_3OS$ $(M+H)^+$: 286.1; found 286.2.

^1H NMR (600 MHz, $CDCl_3$) δ 8.05 (s, 1H), 7.93 (d, *J* = 8.2 Hz, 1H), 7.57 (d, *J* = 8.2 Hz, 1H), 2.24-2.17 (m, 2H), 1.81-1.79 (m, 4H), 1.71-1.66 (m, 2H), 1.42 (s, 3H).

^{13}C NMR (150 MHz, $CDCl_3$) δ 177.2, 160.7, 146.8, 136.6, 127.2, 126.7, 124.2, 122.6, 118.7, 110.3, 50.4, 37.6, 25.0, 25.0.

Biological assays

Radioligand binding assays. HEK293 cells expressing the human A_1- or A_{2A}ARs were cultured in DMEM supplemented with 10% fetal bovine serum, 100 Units/ml penicillin, 100 µg/ml streptomycin, and 2 µmol/ml glutamine. Cells were detached from plates by scraping into cold PBS and centrifuged at 250 *g* for 5 min. The pellets were resuspended in ice-cold Tris HCl buffer (50 mM, pH 7.4) and then homogenized. After homogenization and suspension, cells were centrifuged at 1000 *g* for 10 min, and the pellet was discarded. The suspension was then re-centrifuged at 20,000 *g* for 60 min at 4 °C. The pellets were resuspended in buffer containing 3 Units/ml adenosine deaminase and incubated at 37°C for 30 min. The aliquots of membrane preparations were stored at −80°C until the binding experiments. For displacement binding assays, membrane preparations (20 µg proteins/tube) were incubated at 25°C for 60 min with a final concentration of [^{3}H]DPCPX (0.5 nM) and [^{3}H]ZM241385 (1.0 nM) for A_1- and A_{2A}ARs, respectively, in a mixture containing 50 µL of increasing concentrations of a test ligand in a total assay volume of 200 µL of 50 mM Tris HCl, pH 7.4, containing 10 mM $MgCl_2$. Nonspecific binding was determined using 100 µM of XAC. The reaction was terminated by filtration with GF/B filters using a Brandel 24-channel harvester. Filters were placed in scintillation vials containing 5 ml of Hydrofluor scintillation buffer and counted using a PerkinElmer Tricarb 2810TR liquid scintillation counter.

cAMP assay. HEK293 cells were grown in DMEM supplemented with 10% fetal bovine serum, 100 Units/ml penicillin, 100 µg/ml streptomycin, and 2 µmol/ml glutamine. After 24 hours, cells were treated with assay buffer containing rolipram (10 µM), PSB603 (1 µM) and adenosine deaminase (3 units/ml) for 30 min followed by the addition of antagonists for 20 min and subsequent addition of agonists and incubated for another 20 min. For the A_1AR assay, after incubation with agonist for 20 min, forskolin (10 µM) was added and incubated for an additional 15 min. The reaction was terminated upon removal of the supernatant, and addition of 100 µl Tween-20 (0.3%). Intracellular cAMP levels

were measured with an ALPHAScreen cAMP assay kit following the instructions from the manufacturer (PerkinElmer).

Supplementary Figures

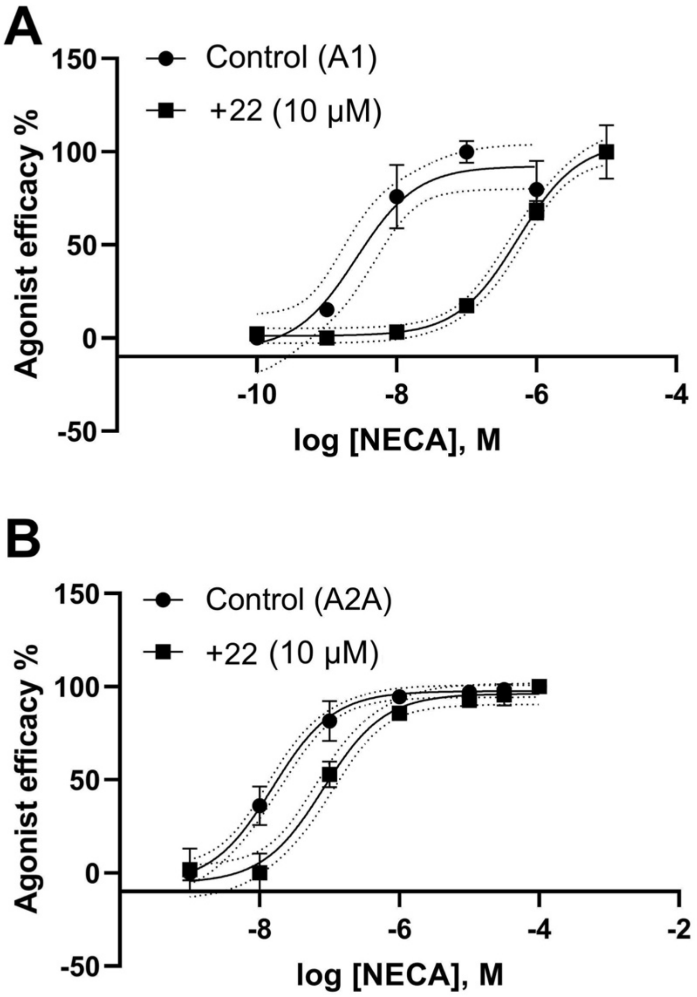

Figure S1. Antagonism by compound **22** of A_1- and A_{2A}AR function demonstrated using a cAMP assay (n=3).

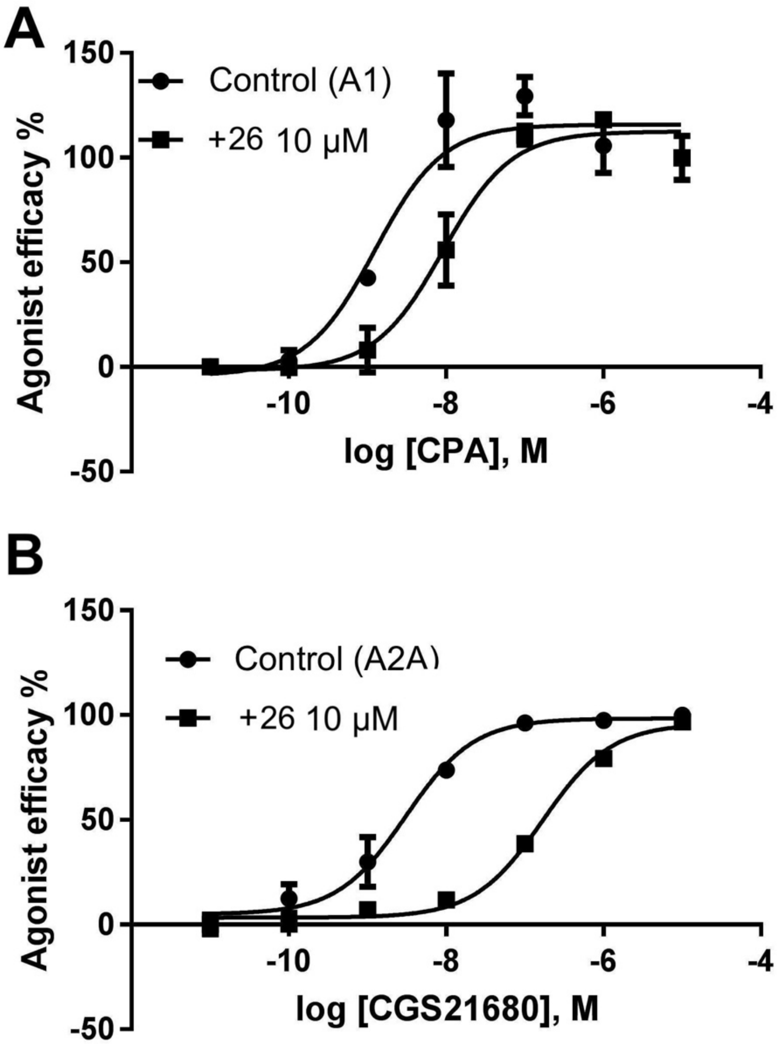

Figure S2. Antagonism by compound **26** of A_1- and A_{2A}AR function demonstrated using a cAMP assay (n=3).

Supplementary Tables

Table S1. Binding affinities of compounds **1**-**10** based on displacement of [^{3}H]DPCPX and [^{3}H]ZM241385 at the A_1AR and $A_{2A}AR$, respectively.

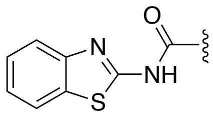

Compound	Substituent	K_i (μM)[a] (ΔG/HA)[b]		A_1AR selectivity index
		A_1AR	$A_{2A}AR$	
**1**	CH_3	11.2 ± 2.4[c] (−6.8/13)	79.1 ± 15.2[c] (−5.6/13)	7.1
**2**	CH_2CH_3	3.46 ± 1.48 (−7.5/14)	6.19 ± 3.43 (−7.1/14)	1.8
**3**	$CH(CH_3)_2$	1.42 ± 0.62 (−8.0./15)	2.26 ± 0.36 (−7.7/15)	1.6
**4**	$_cC_3H_5$	2.35 ± 0.62 (−7.7/15)	3.27 ± 1.14 (−7.5/15)	1.4
**5**	$C(CH_3)_3$	0.285 ± 0.053 (−9.0/16)	1.43 ± 0.23 (−8.0/16)	5.0
**6**	$CH(CH_2CH_3)_2$	1.04 ± 0.10 (−8.2/17)	2.92 ± 0.72 (−7.6/17)	2.8
**7**	$_cC_6H_{11}$	1.08 ± 0.28 (−8.2/18)	3.41 ± 0.95 (−7.5/18)	3.2
**8**	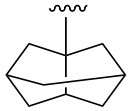	0.484 ± 0.173 (−8.7/21)	1.94 ± 0.22 (−7.8/21)	4.0
**9**	$_cC_5H_9$	0.619 ± 0.141 (−8.5/17)	1.61 ± 0.11 (−7.9/17)	2.6
**10**	$_cC_6H_5$	0.670 ± 0.080 (−8.5/18)	0.386 ± 0.043 (−8.8/18)	0.6

[a] K_i values are expressed as means ± SEM from (2-4) independent experiments.
[b] Free energy of binding (ΔG) calculated from experimental binding affinities (kcal/mol) / Number of heavy atoms (HA). The ligand efficiency (LE) = −ΔG/HA.
[c] Values from Matricon *et al.*[8]

Table S2. Experimental and calculated relative binding free energies at the A_1AR for compounds **2**-**10** with compound **1** as reference.

Compound	ΔΔG (kcal/mol)	
	Experimental[a]	Predicted[b]
**2**	−0.7 ± 0.3	−0.4 ± 0.1
**3**	−1.2 ± 0.3	−1.4 ± 0.1
**4**	−0.9 ± 0.2	−1.2 ± 0.1
**5**	−2.2 ± 0.2	−2.0 ± 0.2
**6**	−1.4 ± 0.1	−2.9 ± 0.2
**7**	−1.4 ± 0.2	−2.8 ± 0.5
**8**	−1.9 ± 0.3	−3.5 ± 0.4
**9**	−1.7 ± 0.2	−2.6 ± 0.2
**10**	−1.7 ± 0.1	−2.2 ± 0.2

[a] Based on Table S1.

[b] Based on three independent sets of MD simulations. Values are expressed as means ± SD.

Table S3. Binding affinities of compounds **11**-**26** based on displacement of [^{3}H]DPCPX and [^{3}H]ZM241385 at the A_1AR and A_{2A}AR, respectively.

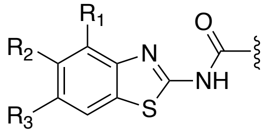

ID	Substituent	R_1	R_2	R_3	K_i (μM)[a] (ΔG/HA)[b]		A_1AR selectivity index
					A_1AR	A_{2A}AR	
**11**		-	-	CH_3	> 100 (> −5.5/17)	25.8 ± 13.2 (−6.3/17)	< 0.3
**12**		CH_3	-	-	1.04 ± 0.14 (−8.2/17)	6.75 ± 0.92 (−7.1/17)	6.5
**13**		-	CH_3	-	0.076 ± 0.017 (−9.8/17)	1.45 ± 0.60 (−8.0/17)	19.1
**14**		-	Cl	-	0.117 ± 0.011 (−9.5/17)	1.29 ± 0.28 (−8.1/17)	11.0
**15**	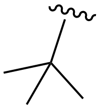	-	Br	-	0.040 ± 0.010 (−10.2/17)	0.746 ± 0.149 (−8.4/17)	18.7
**16**		-	CH_2CH_3	-	0.143 ± 0.02 (−9.4/18)	3.38 ± 0.53 (−7.5/18)	23.6
**17**		-	CN	-	0.223 ± 0.022 (−9.1/18)	5.63 ± 0.74 (−7.2/18)	25.2
**18**		-	OCH_3	-	0.152 ± 0.057 (−9.4/18)	1.60 ± 0.51 (−8.0/18)	10.5
**19**		OCH_3	-	-	0.133 ± 0.01 (−9.4/18)	0.199 ± 0.07 (−9.2/18)	1.5
**20**		-	-	-	0.095 ± 0.008 (−9.6/18)	0.572 ± 0.064 (−8.6/18)	6.0
**21**		-	Cl	-	0.023 ± 0.006 (−10.5/19)	0.252 ± 0.089 (−9.1/19)	11.0
**22**	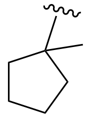	-	Br	-	0.010 ± 0.008 (−11.0/19)	0.384 ± 0.082 (−8.8/19)	38.4
**23**		-	CH_3	-	0.057 ± 0.015 (−9.9/19)	0.561 ± 0.109 (−8.6/19)	9.8
**24**		-	CH_2CH_3	-	0.096 ± 0.013 (−9.6/20)	2.15 ± 0.09 (−7.8/20)	22.4
**25**		-	CN	-	0.064 ± 0.006 (−9.9/20)	1.78 ± 0.74 (−7.9/20)	27.8
**26**	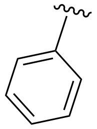	OCH_3	-	-	1.33 ± 0.59 (−8.1/20)	0.106 ± 0.020 (−9.6/20)	0.1

[a] K_i values are expressed as means ± SEM from (2-4) independent experiments.
[b] Free energy of binding (ΔG) calculated from experimental binding affinities (kcal/mol) / Number of heavy atoms (HA). The ligand efficiency (LE) = −ΔG/HA.

Table S4. Experimental and calculated relative binding free energies at the A_1AR and $A_{2A}AR$ for compounds **11**-**26**.

Compound pair		ΔΔG (kcal/mol)			
		A_1AR		$A_{2A}AR$	
**ID**	**Reference**	**Experimental[a]**	**Predicted[b]**	**Experimental[a]**	**Predicted[b]**
**11**	**5**	> 3.5	0.2 ± 0.1	1.7 ± 0.6	1.7 ± 0.1
**12**		0.8 ± 0.1	−0.7 ± 0.1	0.9 ± 0.1	−0.9 ± 0.1
**13**		−0.8 ± 0.2	−1.8 ± 0.1	0.0 ± 0.5	−1.6 ± 0.1
**14**		−0.5 ± 0.1	−1.6 ± 0.1	−0.1 ± 0.2	−1.2 ± 0.2
**15**		−1.2 ± 0.2	−2.0 ± 0.1	−0.4 ± 0.2	−1.3 ± 0.1
**16**		−0.4 ± 0.1	−1.6 ± 0.1	0.5 ± 0.4	−0.7 ± 0.1
**17**		−0.2 ± 0.1	−0.8 ± 0.1	0.8 ± 0.1	0.3 ± 0.1
**18**		−0.4 ± 0.3	−1.7 ± 0.2	0.1 ± 0.5	−2.2 ± 0.2
**19**		−0.5 ± 0.1	−1.2 ± 0.3	−1.2 ± 0.2	−2.4 ± 0.3
**20**	**9**	−1.1 ± 0.1	−2.3 ± 0.1	−0.6 ± 0.1	−0.6 ± 0.1
**21**		−2.0 ± 0.2	−3.4 ± 0.1	−1.1 ± 0.2	−1.8 ± 0.1
**22**		−2.4 ± 0.1	−3.7 ± 0.1	−0.8 ± 0.1	−1.8 ± 0.1
**23**		−1.4 ± 0.2	−3.8 ± 0.1	−0.6 ± 0.1	−2.2 ± 0.1
**24**		−1.1 ± 0.1	−3.4 ± 0.1	0.2 ± 0.1	−1.6 ± 0.2
**25**		−1.3 ± 0.1	−2.7 ± 0.2	0.1 ± 0.3	−0.8 ± 0.1
**26**	**10**	0.4 ± 0.3	1.2 ± 0.4	−0.8 ± 0.1	−0.6 ± 0.3

[a] Based on Table S3.

[b] Based on three independent sets of MD simulations. Values are expressed as means ± SD.

Table S5. Highest affinity human A_1AR ligands (with < 20 HA) in the ChEMBL database compared to compound **22**.[a]

Compound	K_i (nM)		A_1AR selectivity
	hA_1AR	$hA_{2A}AR$	
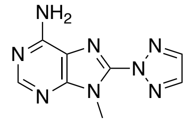 CHEMBL194728	0.4	46.3	115.7
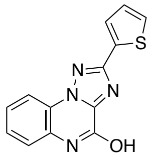 CHEMBL328437	2	-	-
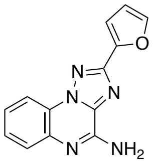 CHEMBL261345	5.6	11.2	2.0
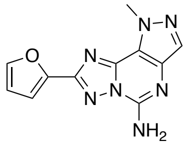 CHEMBL1935752	10	3.6	0.4
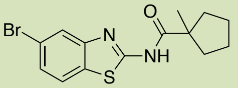 Compound **22**	10	384	38.4
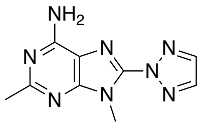 CHEMBL194290	10.4	70.4	6.8

[a] Known A_1AR and $A_{2A}AR$ ligands with determined K_i values were downloaded from the ChEMBL database. Compounds with motifs found in pan-assay interference compounds[16] were excluded using OpenEye Toolkits 2020.2.0 OpenEye Scientific Software, Santa Fe, NM. http://www.eyesopen.com.

NMR spectra

Compounds synthesized by Enamine (**2-6** and **9-10**)

Compound **2**

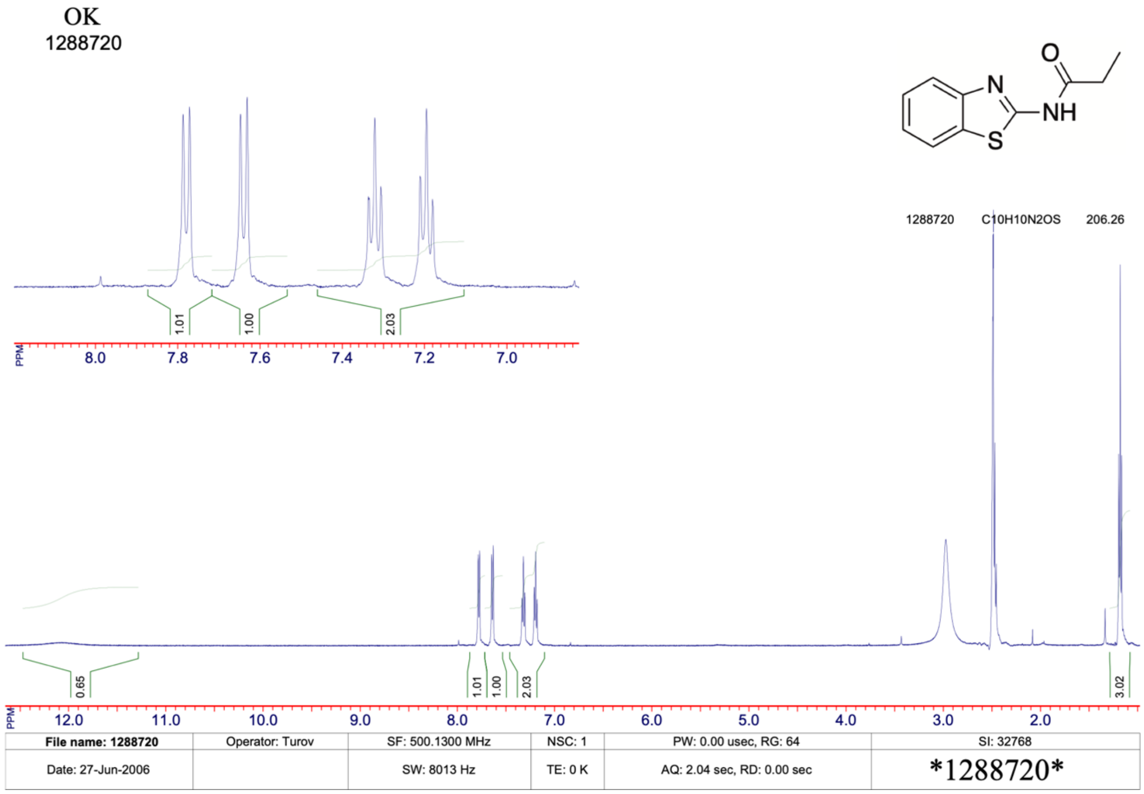

Compound **3**

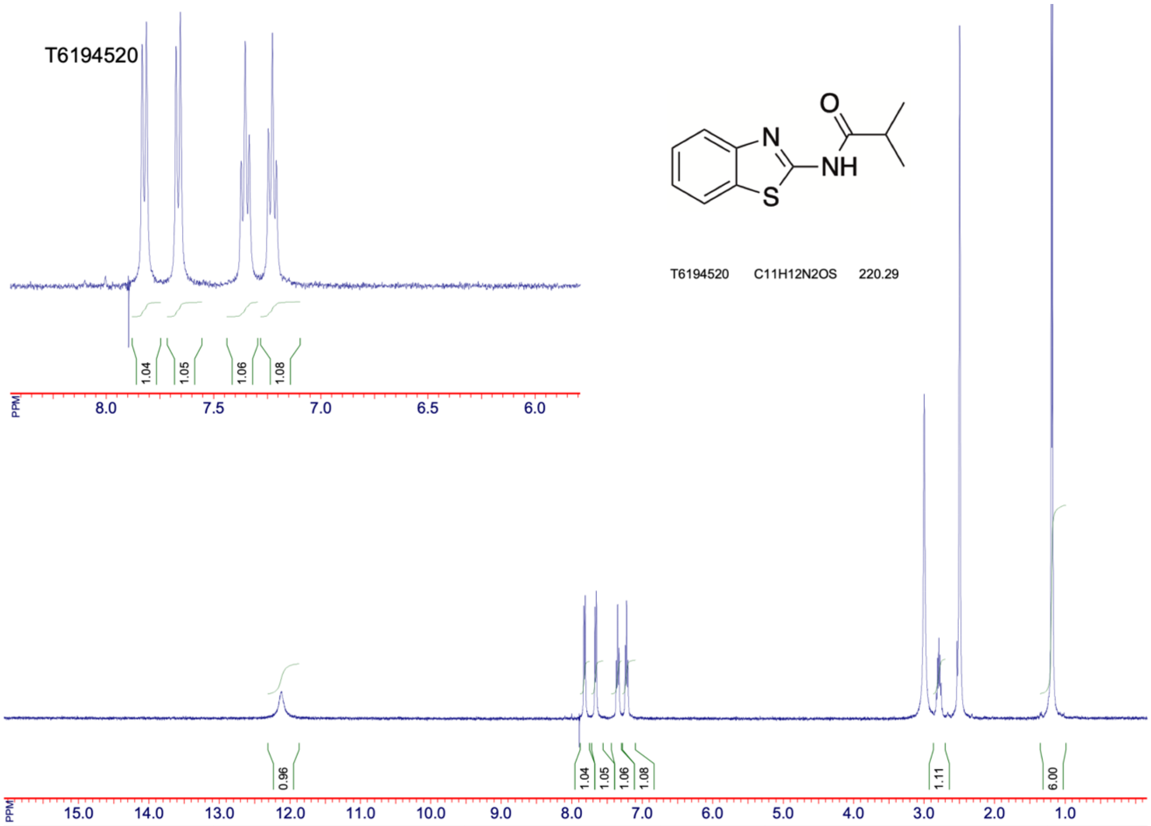

File name: T6194520	Operator: Victorova	SF: 399.9831 MHz	NSC: 0	PW: 7.50 usec, RG: 34	SI: 32768
Date: 08-Aug-2017	Solvent: dmso_d6+CCl4	SW: 7639 Hz	TE: 293 K	AQ: 1.05 sec, RD: 0.00 sec	

Compound **4**

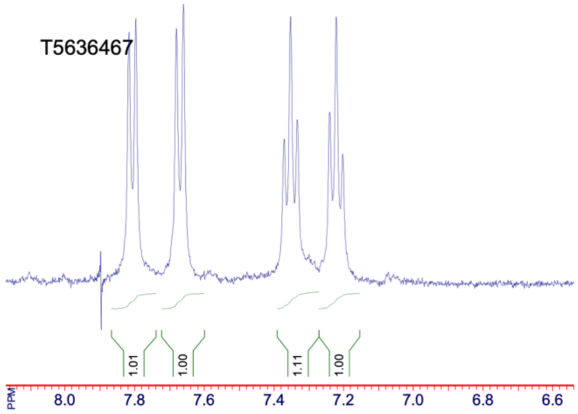

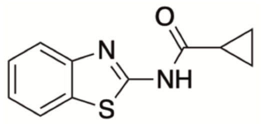

T5636467 C11H10N2OS 218.27

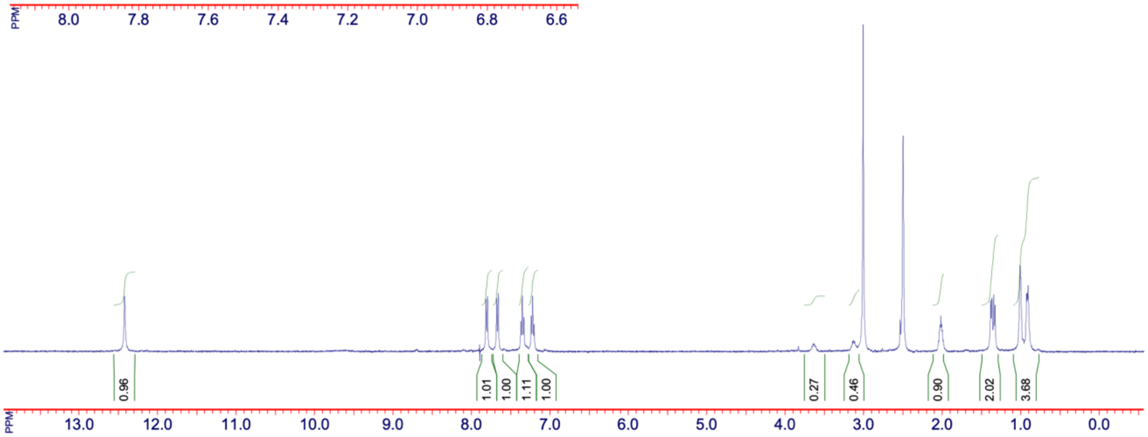

**File name: T5636467**	Operator: Victorova	SF: 399.9831 MHz	NSC: 0	PW: 7.50 usec, RG: 34	SI: 32768
Date: 08-Aug-2017	Solvent: dmso_d6+CCl4	SW: 7639 Hz	TE: 293 K	AQ: 1.05 sec, RD: 0.00 sec	

Compound **5**

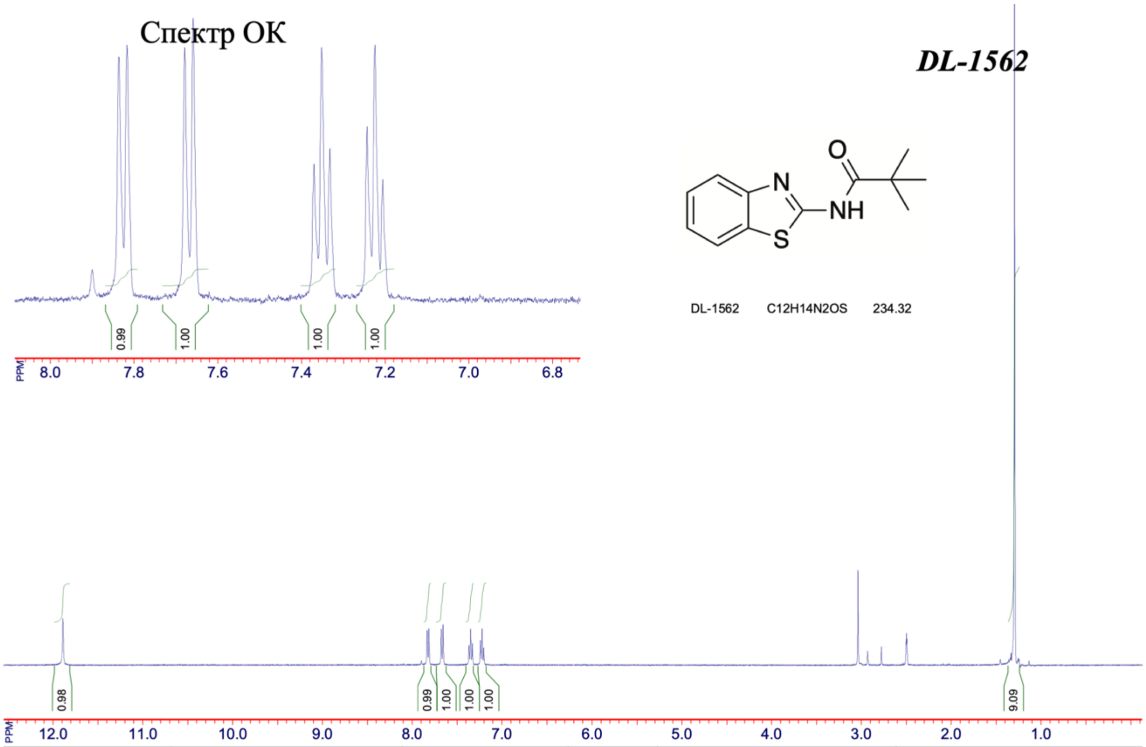

File name: DL-1562	Operator: Reutova	SF: 400.3965 MHz	NSC: 0	PW: 8.00 usec, RG: 38	SI: 65536
Date: 24-Dec-2003	Solvent: DMSO-d6 + CCl4	SW: 8000 Hz	TE: 293 K	AQ: 2.00 sec, RD: 0.00 sec	*DL-1562*

Compound **6**

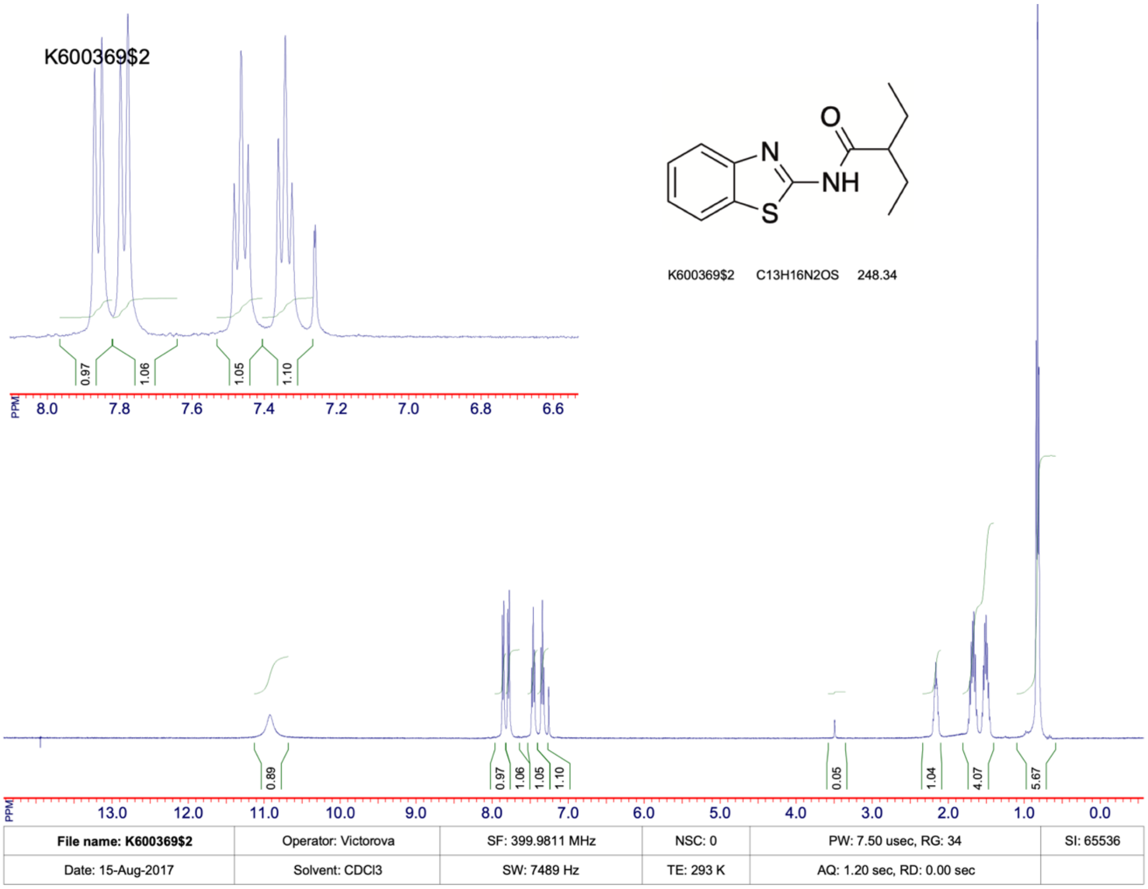

K600369$2
O
N
NH
S
K600369$2 C13H16N2OS 248.34
0.97
1.06
1.05
1.10
PPM 8.0 7.8 7.6 7.4 7.2 7.0 6.8 6.6
0.89
0.97
1.06
1.05
1.10
0.05
1.04
4.07
5.67
PPM 13.0 12.0 11.0 10.0 9.0 8.0 7.0 6.0 5.0 4.0 3.0 2.0 1.0 0.0
File name: K600369$2
Operator: Victorova
SF: 399.9811 MHz
NSC: 0
PW: 7.50 usec, RG: 34
SI: 65536
Date: 15-Aug-2017
Solvent: CDCl3
SW: 7489 Hz
TE: 293 K
AQ: 1.20 sec, RD: 0.00 sec

Compound **9**

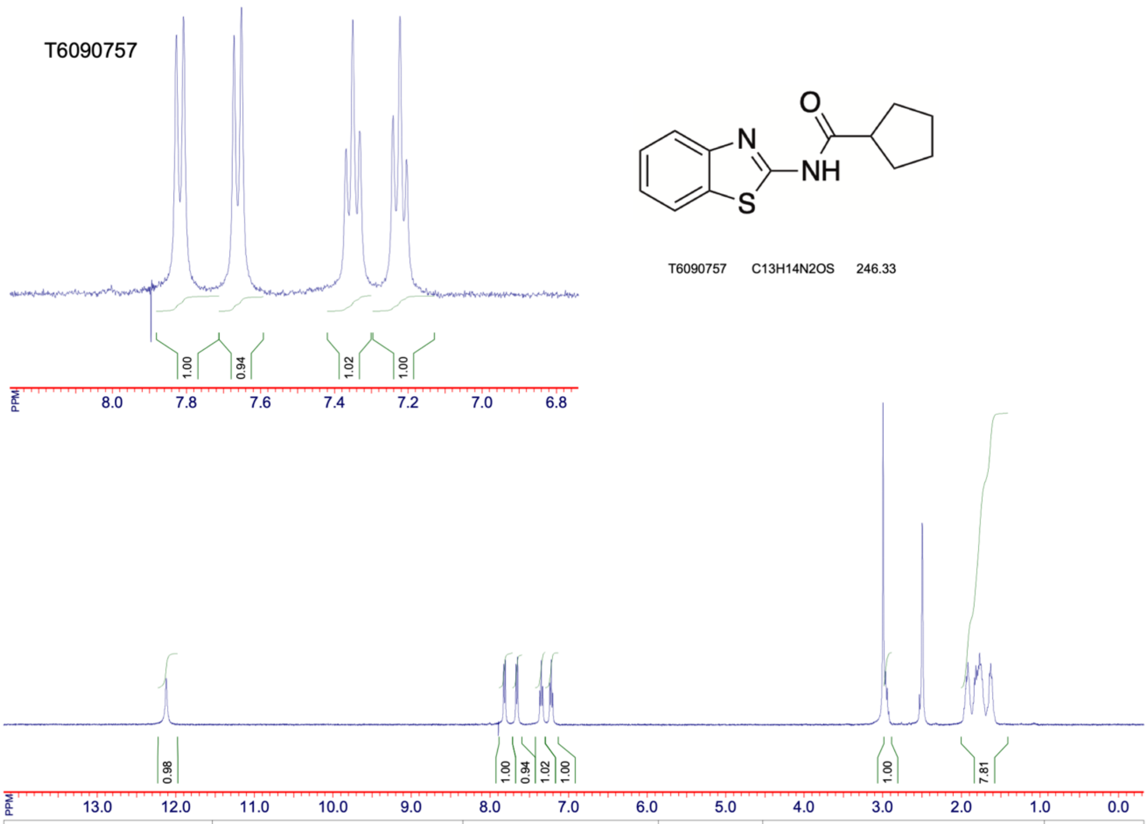

**File name: T6090757**	Operator: Victorova	SF: 399.9831 MHz	NSC: 0	PW: 7.50 usec, RG: 34	SI: 32768
Date: 08-Aug-2017	Solvent: dmso_d6+CCl4	SW: 7639 Hz	TE: 293 K	AQ: 1.05 sec, RD: 0.00 sec	

Compound **10**

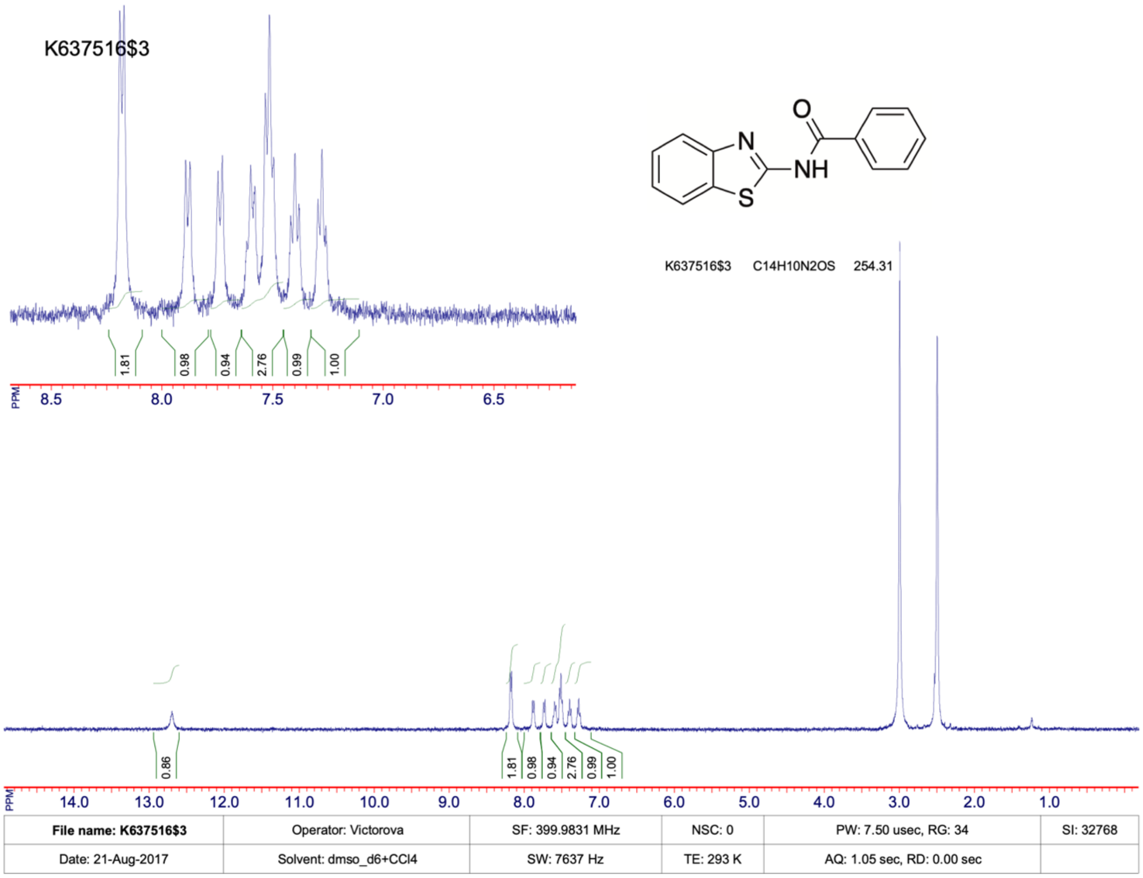
K637516$3
K637516$3 C14H10N2OS 254.31
1.81
0.98
0.94
2.76
0.99
1.00
0.86
PPM
8.5
8.0
7.5
7.0
6.5
14.0
13.0
12.0
11.0
10.0
9.0
6.0
5.0
4.0
3.0
2.0
1.0
File name: K637516$3
Operator: Victorova
SF: 399.9831 MHz
NSC: 0
PW: 7.50 usec, RG: 34
SI: 32768
Date: 21-Aug-2017
Solvent: dmso_d6+CCl4
SW: 7637 Hz
TE: 293 K
AQ: 1.05 sec, RD: 0.00 sec

Synthesized compounds (**7-8** and **11-26**)

^{1}H NMR (500 MHz, $CDCl_3$) of compound **7**

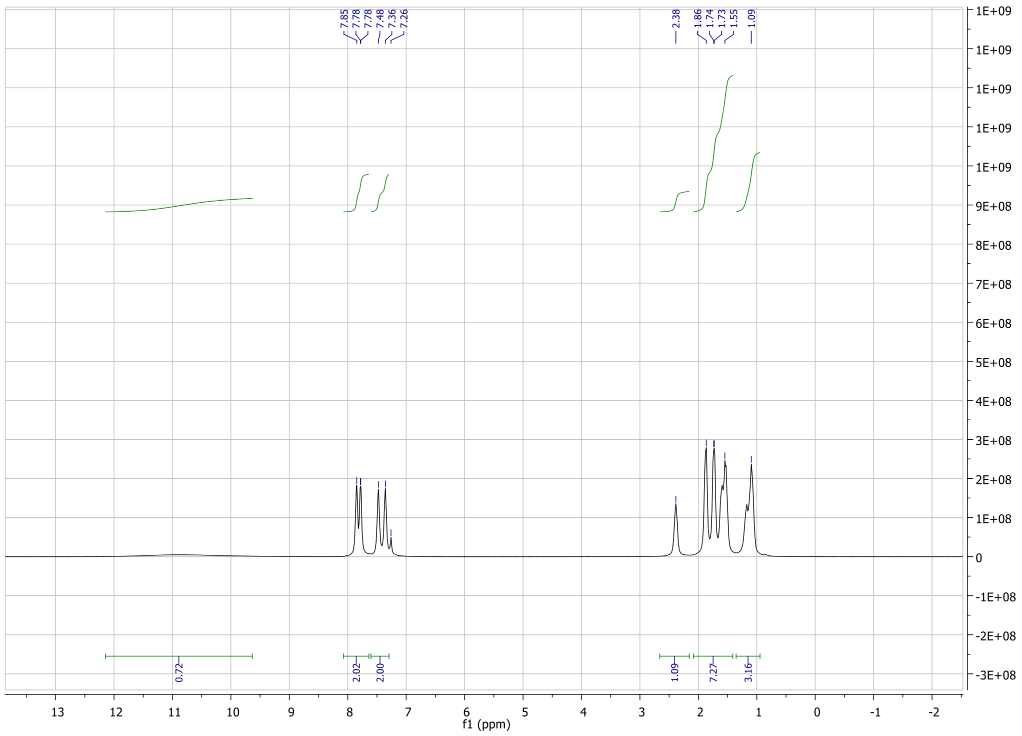

^{13}C NMR (125 MHz, $CDCl_3$) of compound **7**

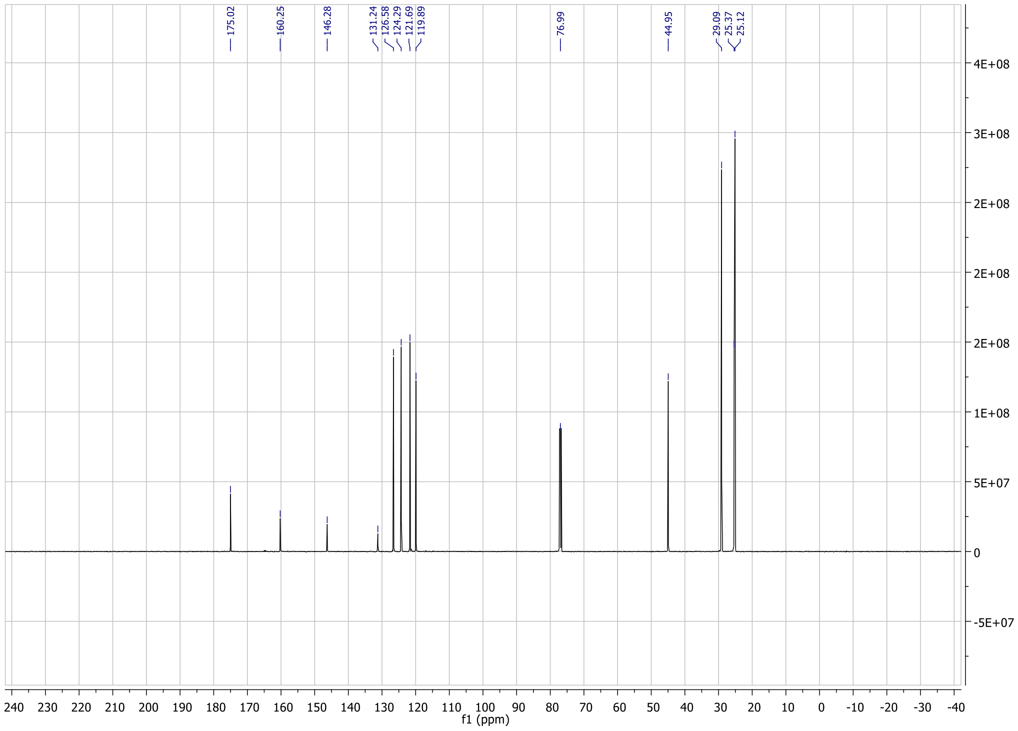

^{1}H NMR (500 MHz, $CDCl_3$) of compound **8**

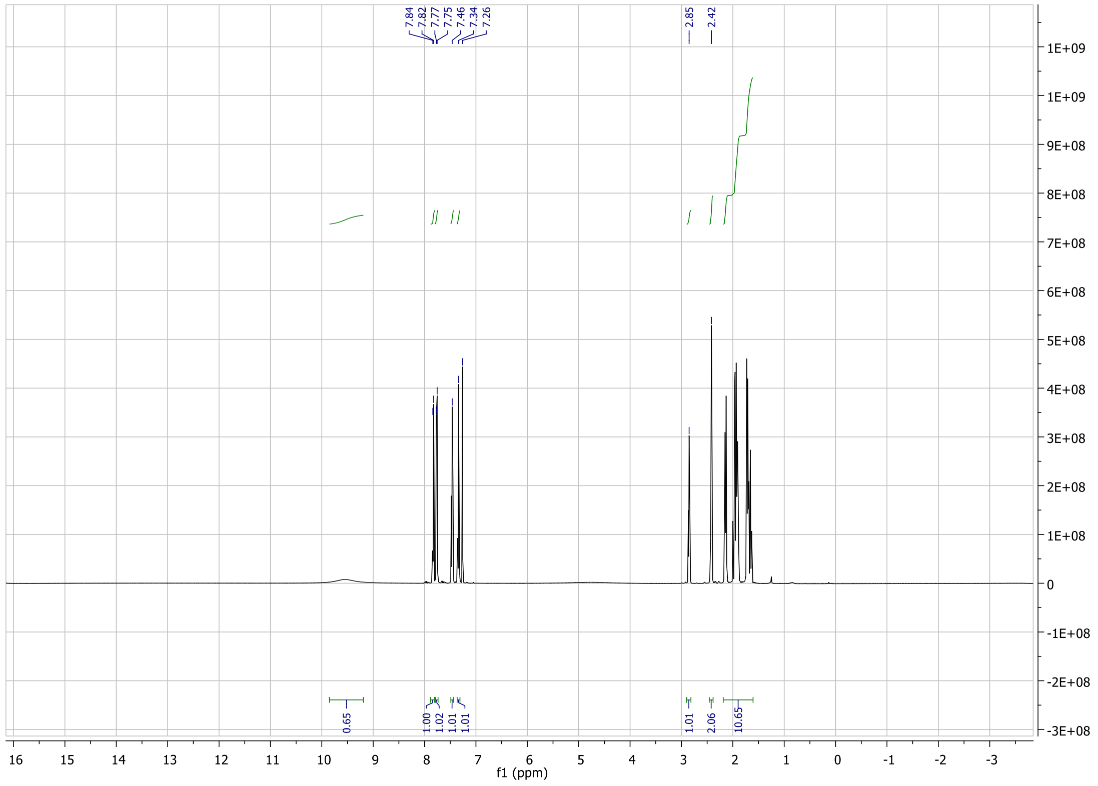

^{13}C NMR (125 MHz, $CDCl_3$) of compound **8**

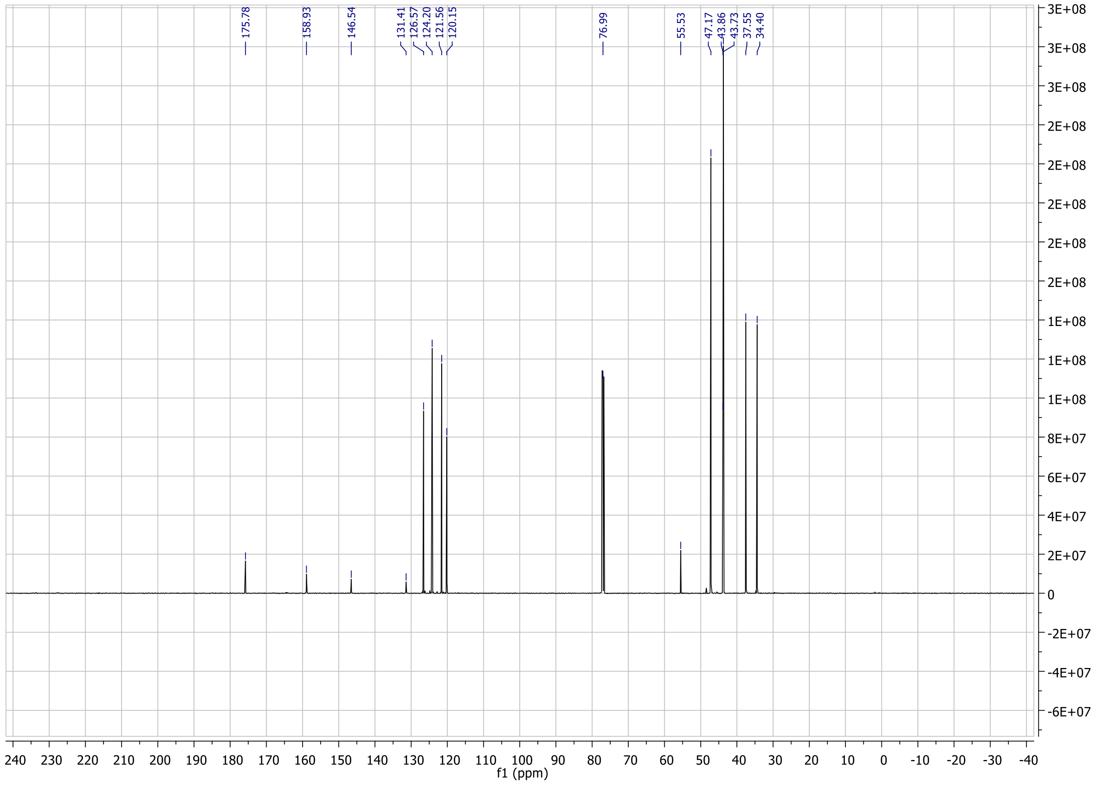

^{1}H NMR (500 MHz, $CDCl_3$) of compound **11**

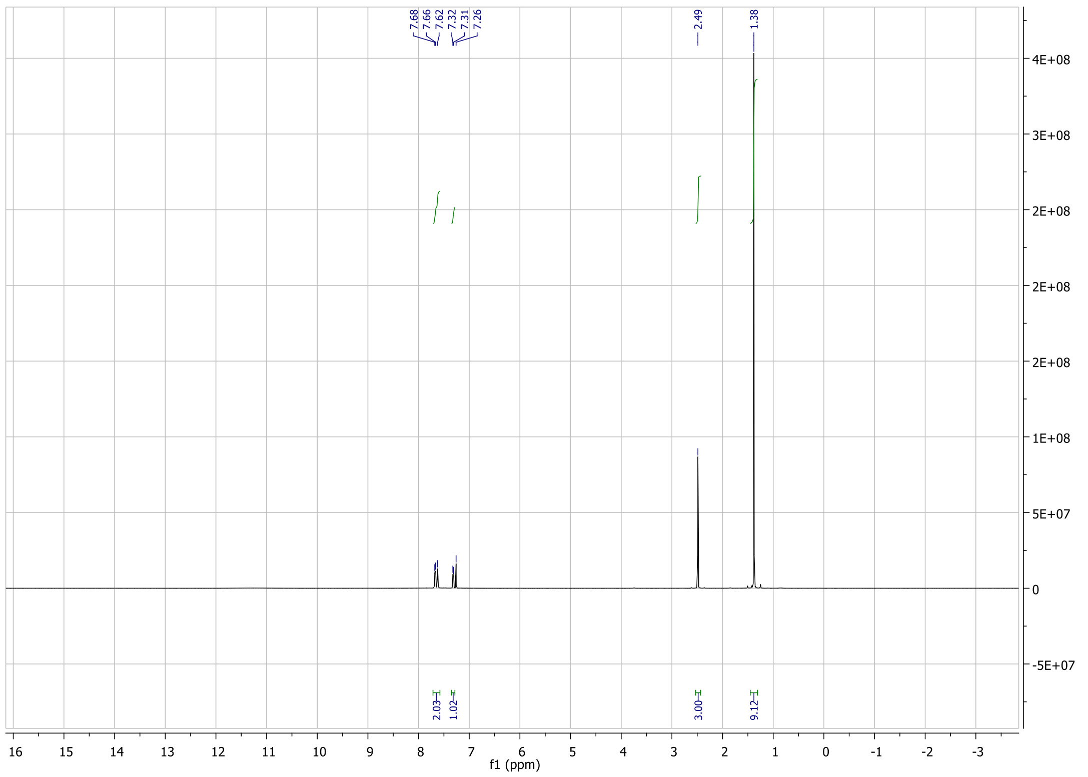

^{13}C NMR (125 MHz, $CDCl_3$) of compound **11**

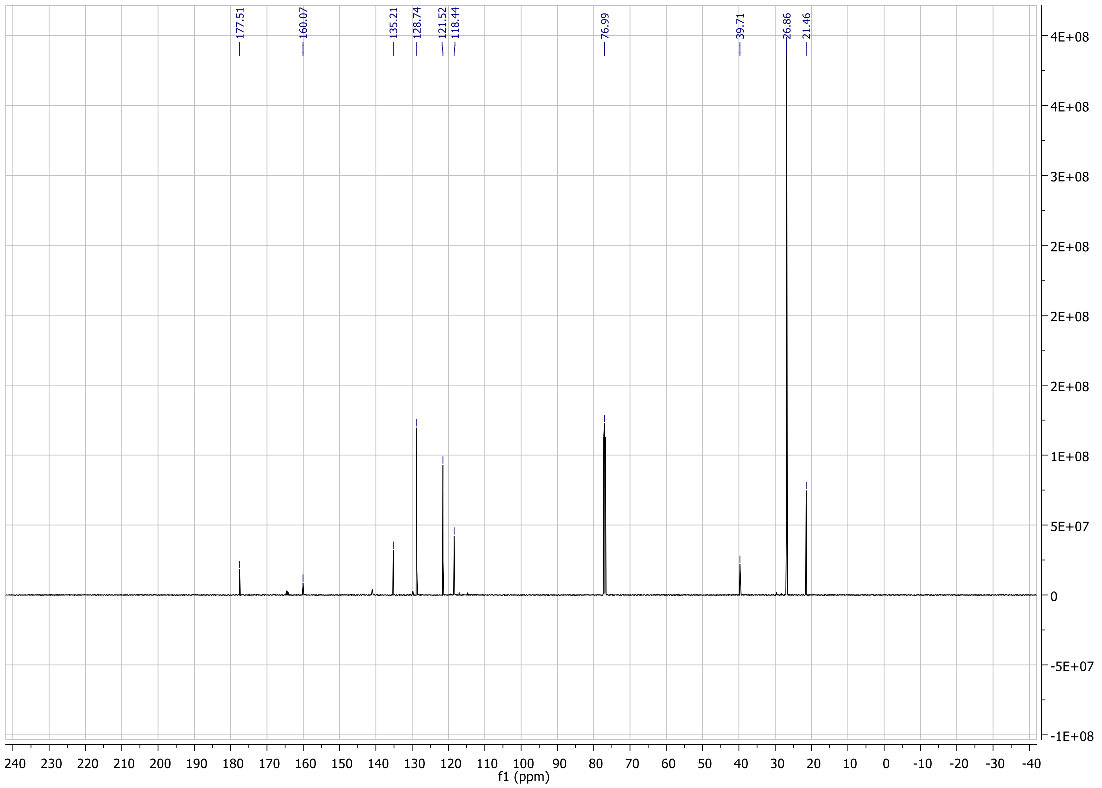

^{1}H NMR (400 MHz, $CDCl_3$) of compound **12**

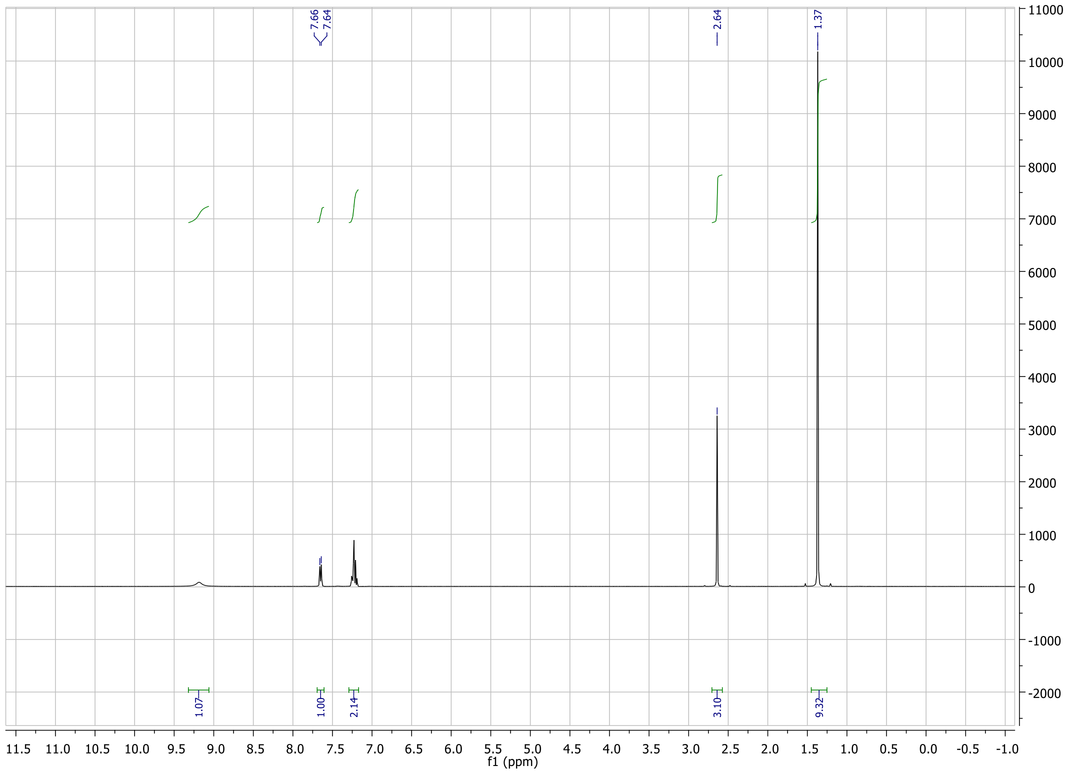

^{13}C NMR (100 MHz, $CDCl_3$) of compound **12**

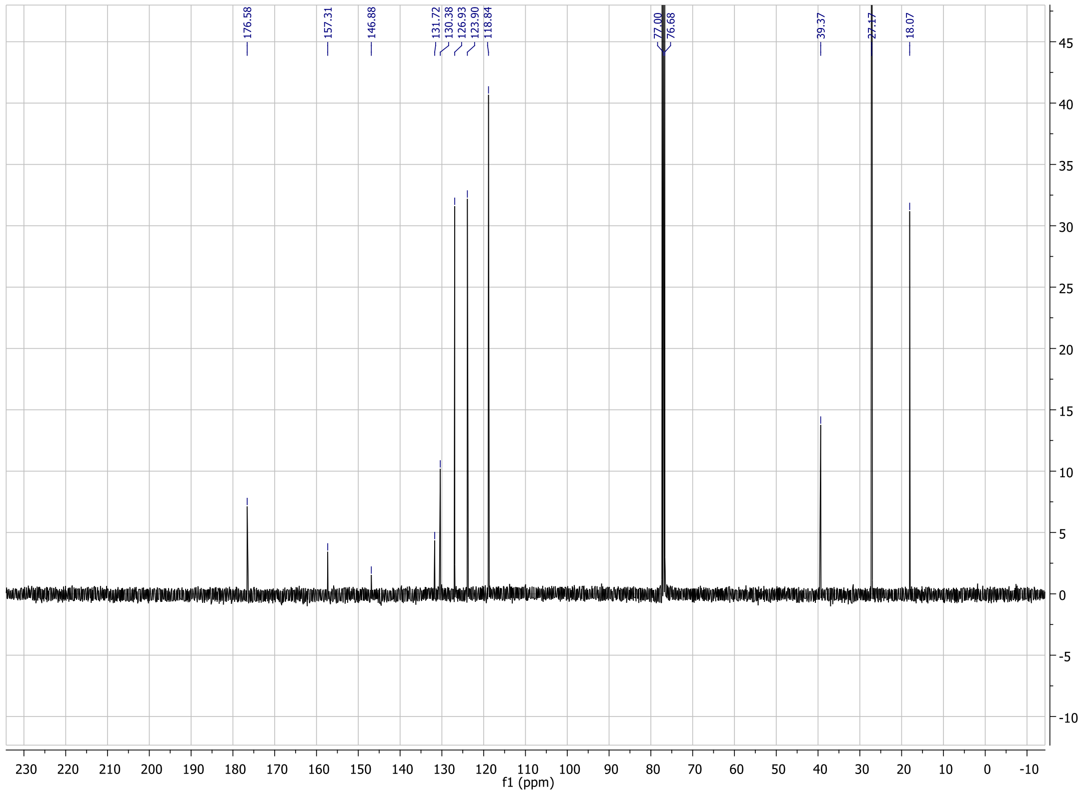

^{1}H NMR (500 MHz, $CDCl_3$) of compound **13**

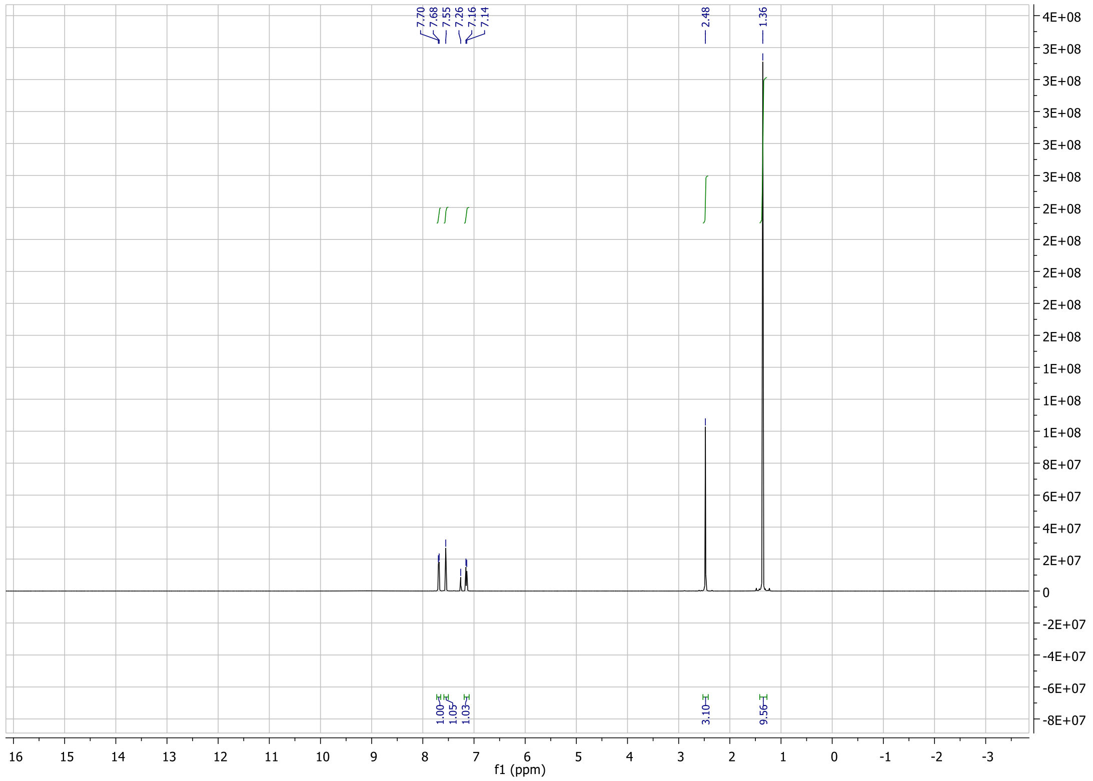

^{13}C NMR (125 MHz, $CDCl_3$) of compound **13**

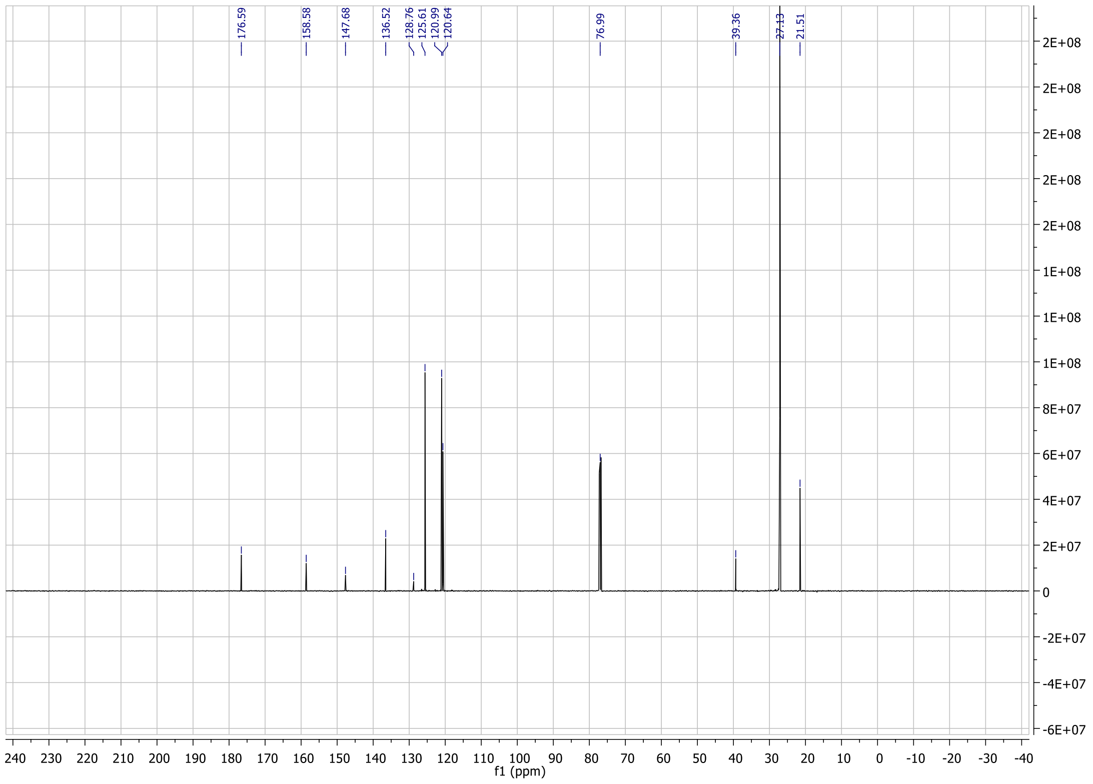

^{1}H NMR (600 MHz, $CDCl_3$) of compound **14**

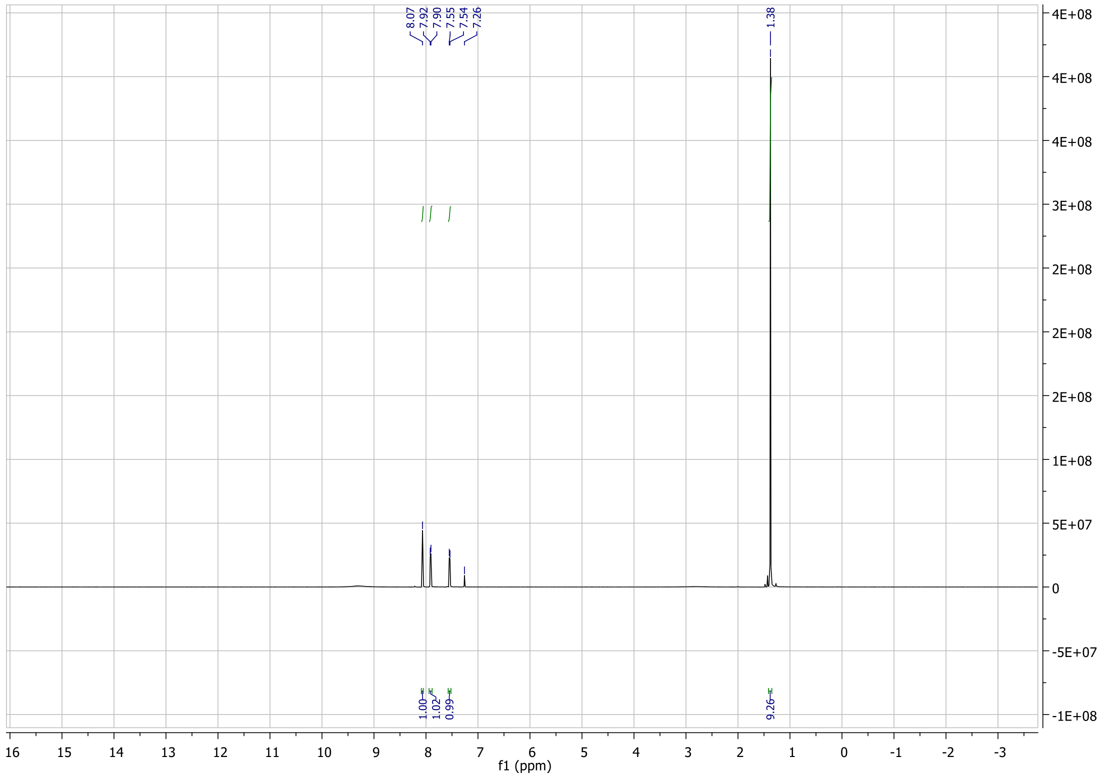

^{13}C NMR (150 MHz, $CDCl_3$) of compound **14**

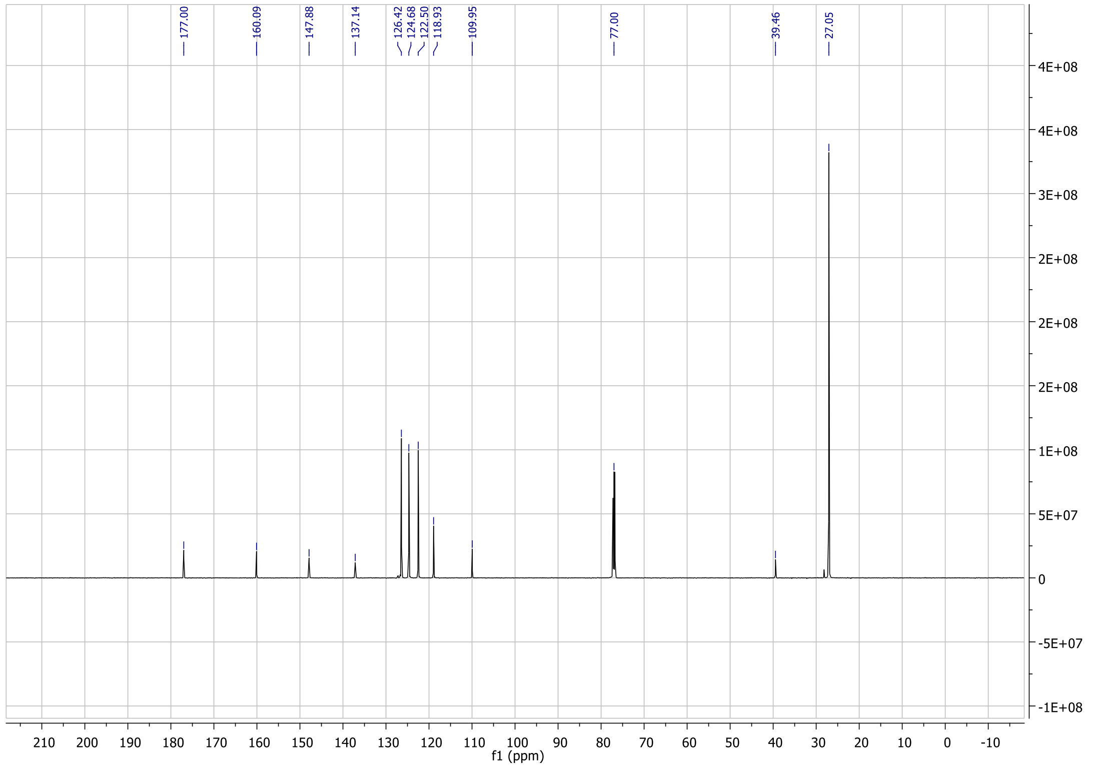

^{1}H NMR (600 MHz, $CDCl_3$) of compound **15**

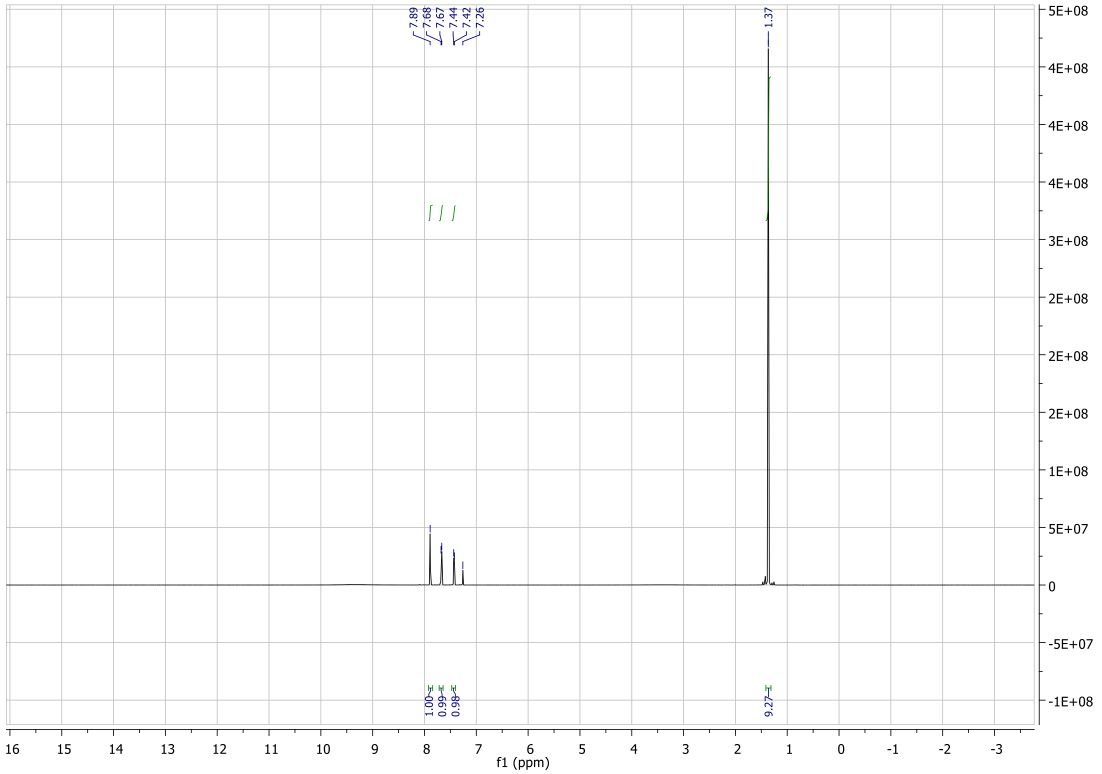

^{13}C NMR (150 MHz, $CDCl_3$) of compound **15**

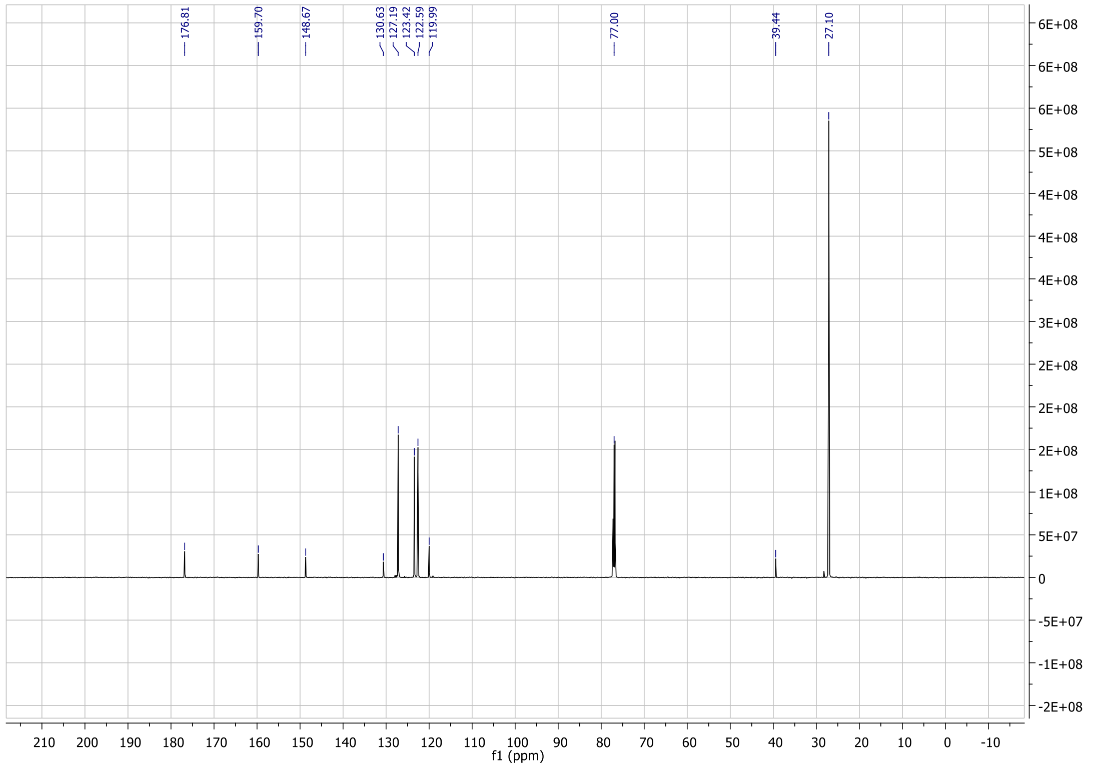

^{1}H NMR (500 MHz, $CDCl_3$) of compound **16**

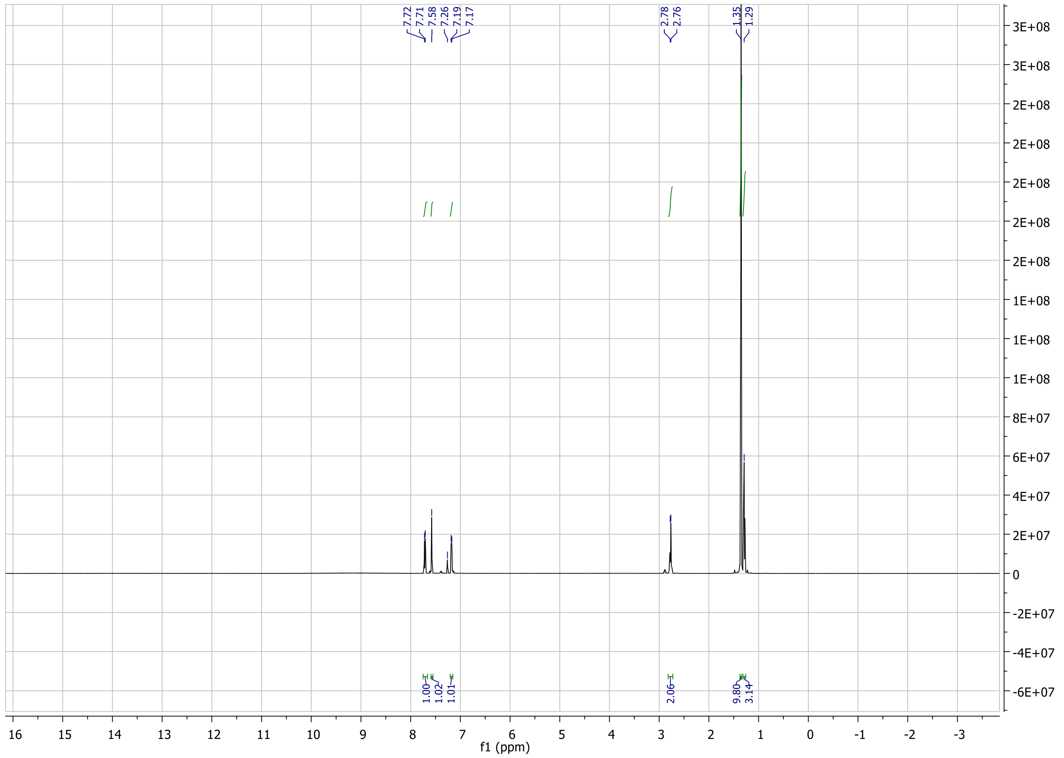

^{13}C NMR (125 MHz, $CDCl_3$) of compound **16**

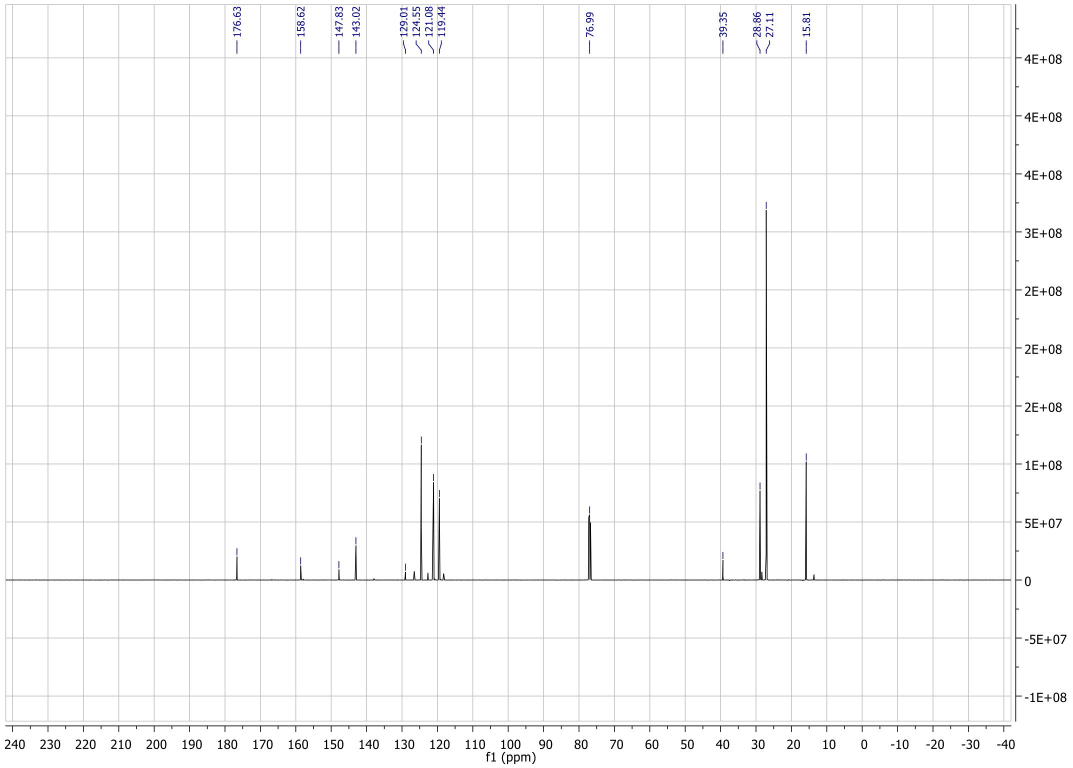

^{1}H NMR (600 MHz, $CDCl_3$) of compound **17**

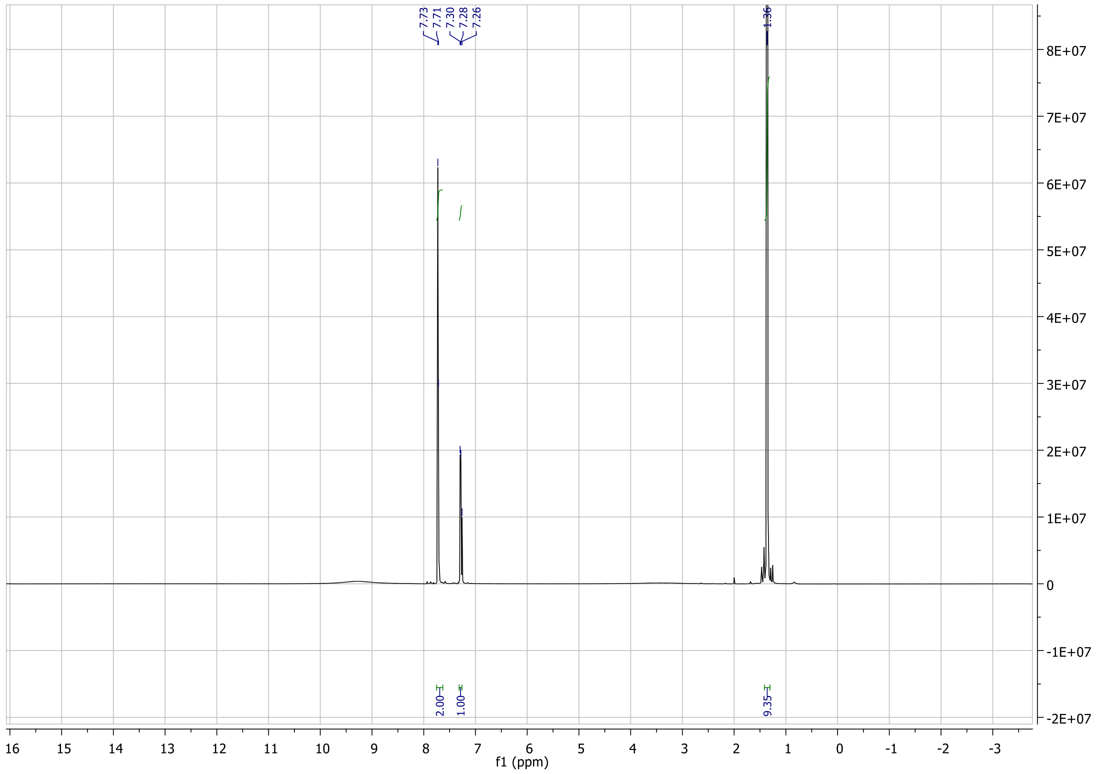

^{13}C NMR (150 MHz, $CDCl_3$) of compound **17**

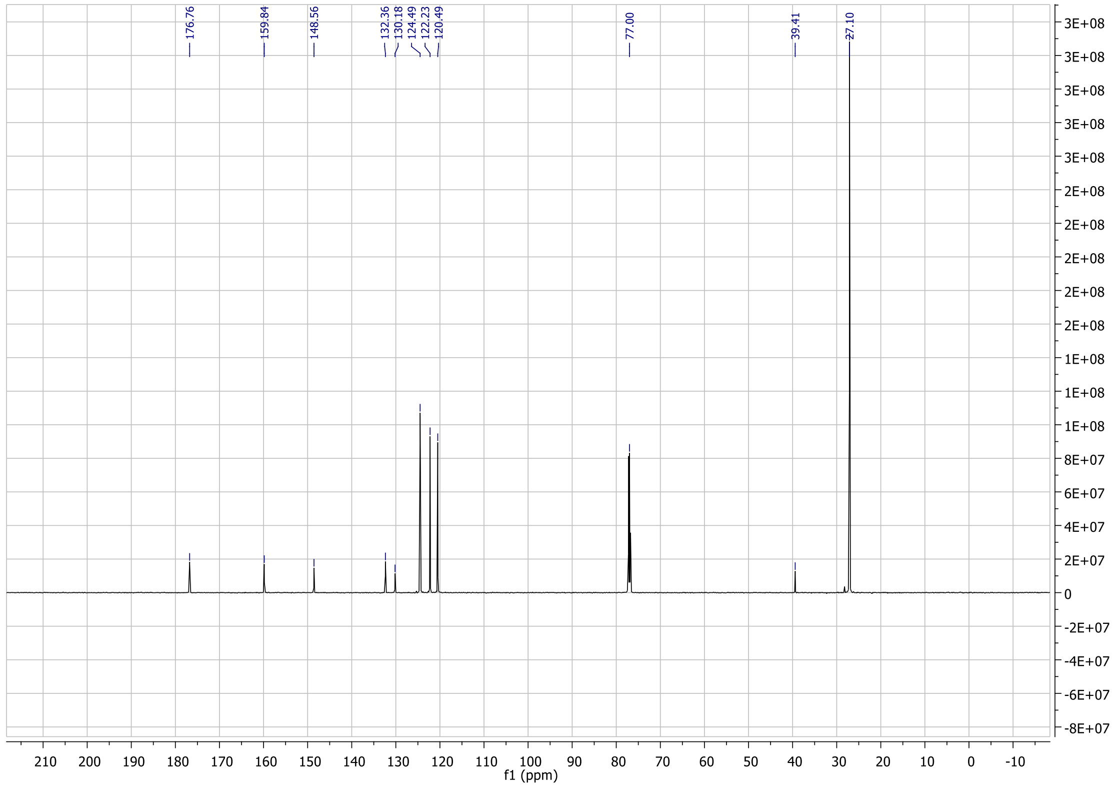

^{1}H NMR (500 MHz, $CDCl_3$) of compound **18**

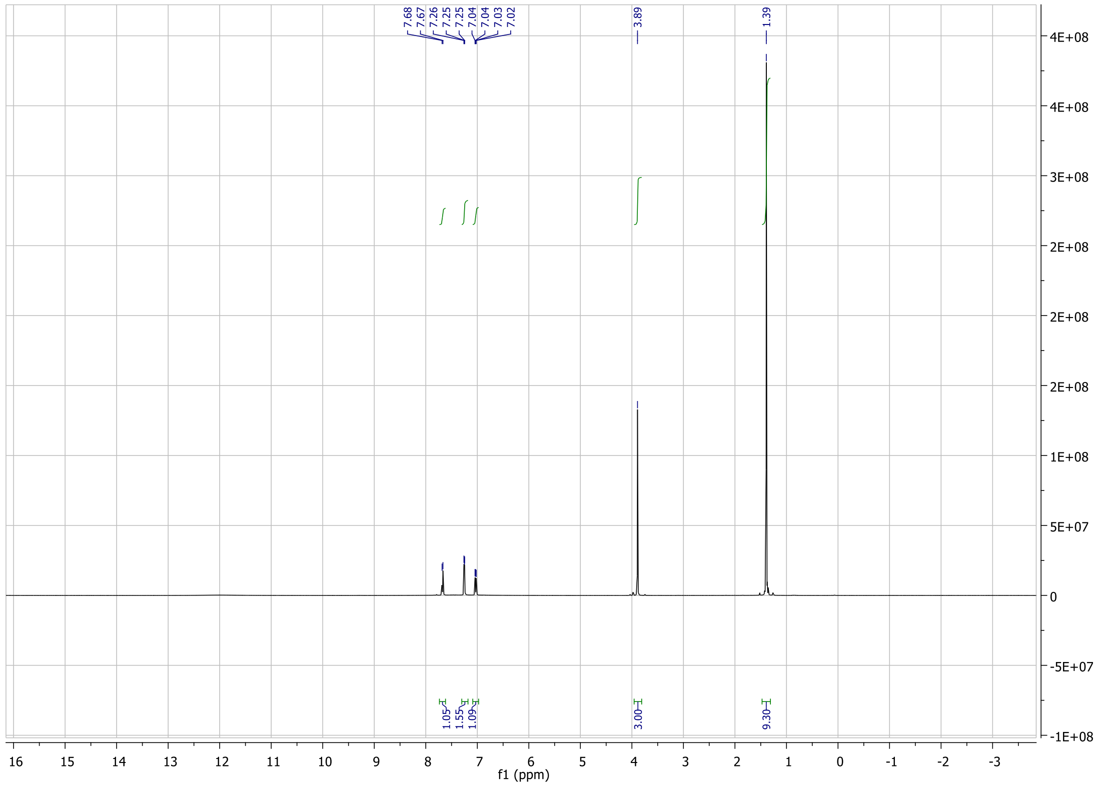

^{13}C NMR (125 MHz, $CDCl_3$) of compound **18**

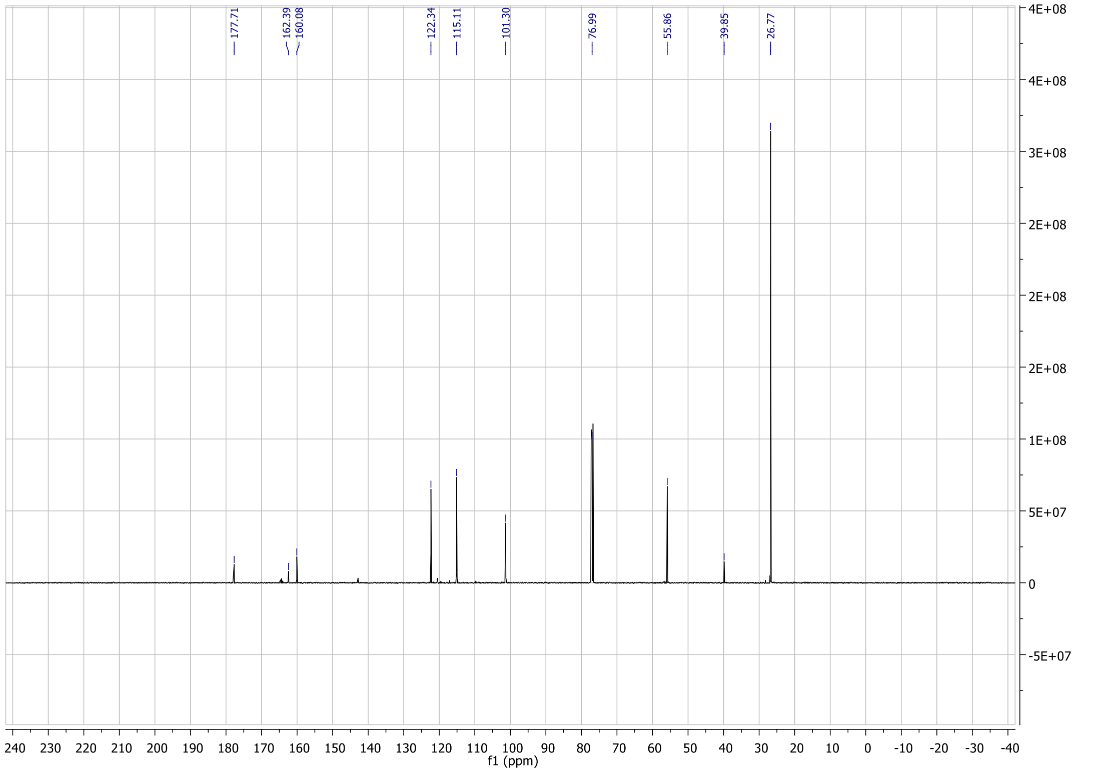

^{1}H NMR (400 MHz, $CDCl_3$) of compound **19**

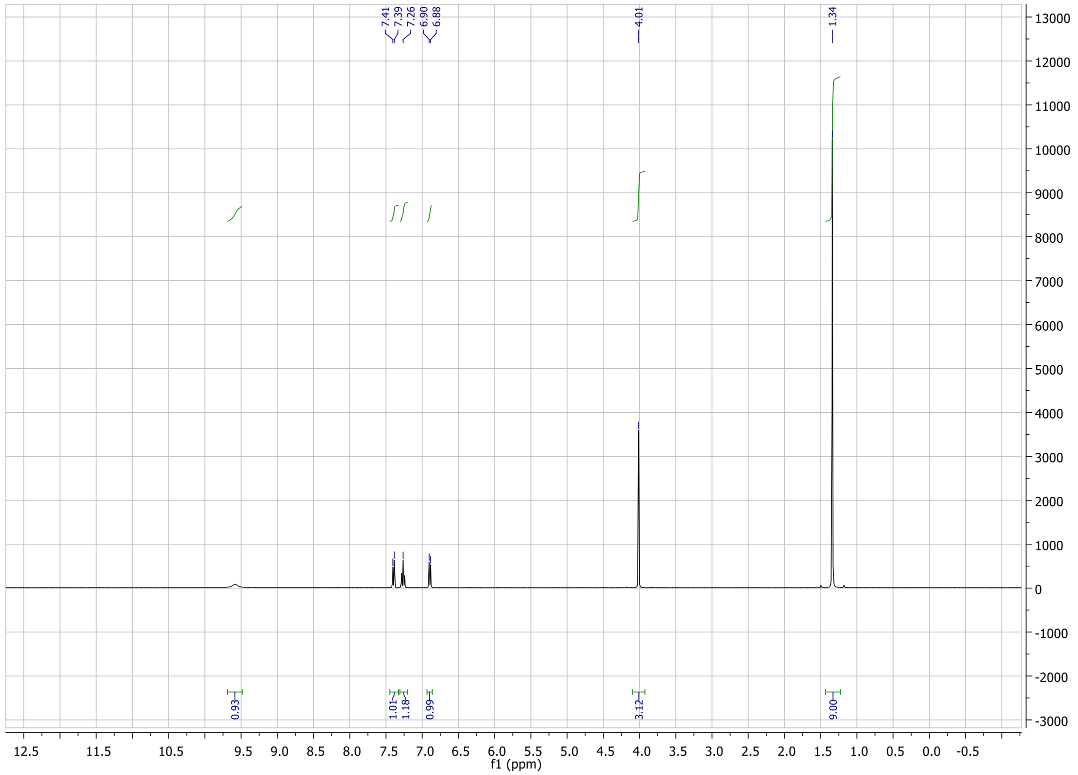

^{13}C NMR (100 MHz, $CDCl_3$) of compound **19**

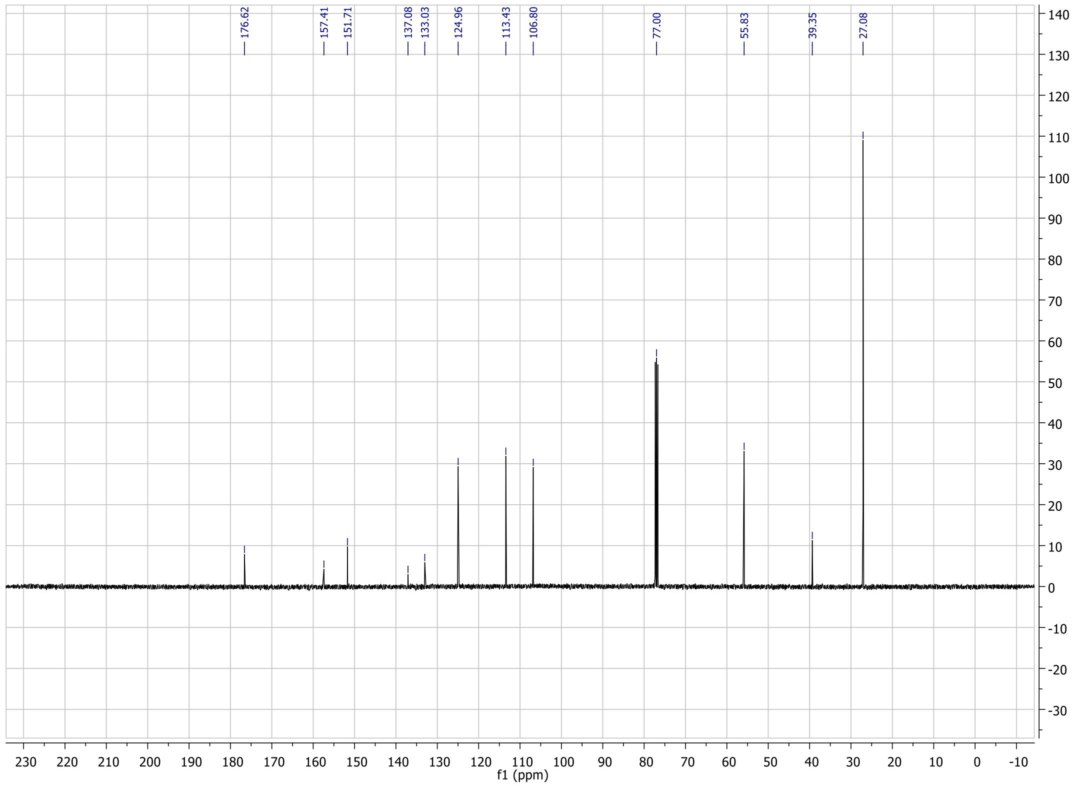

^{1}H NMR (600 MHz, $CDCl_3$) of compound **20**

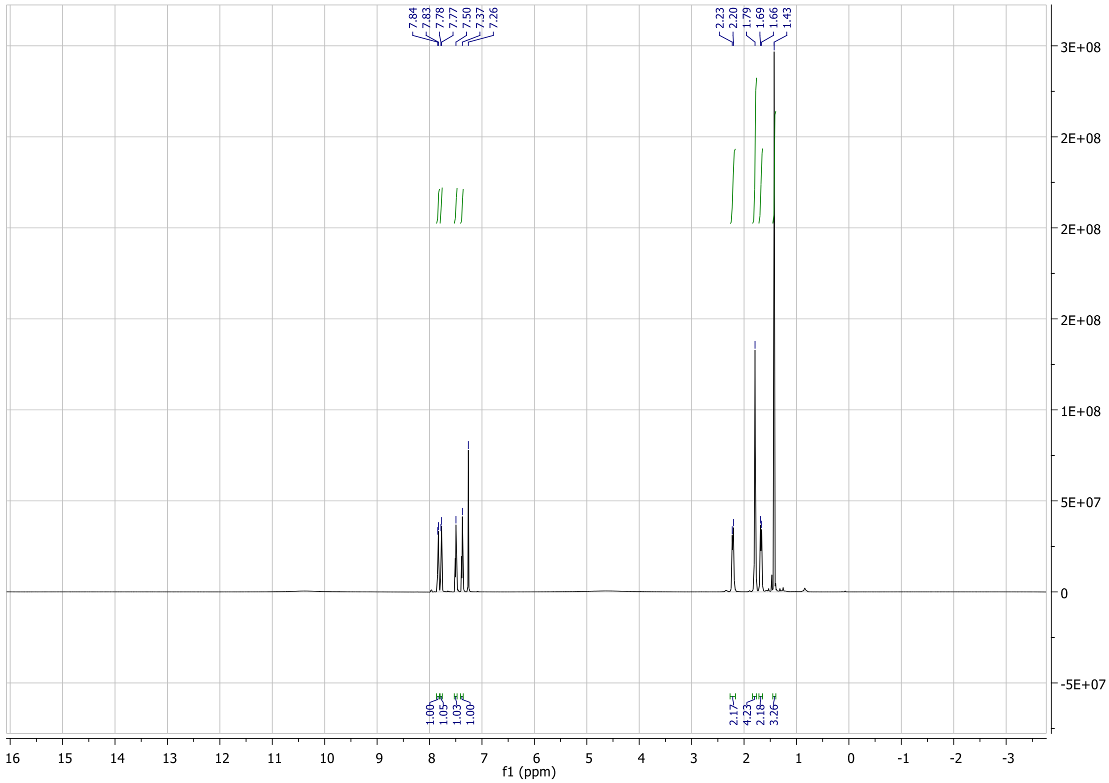

^{13}C NMR (150 MHz, $CDCl_3$) of compound **20**

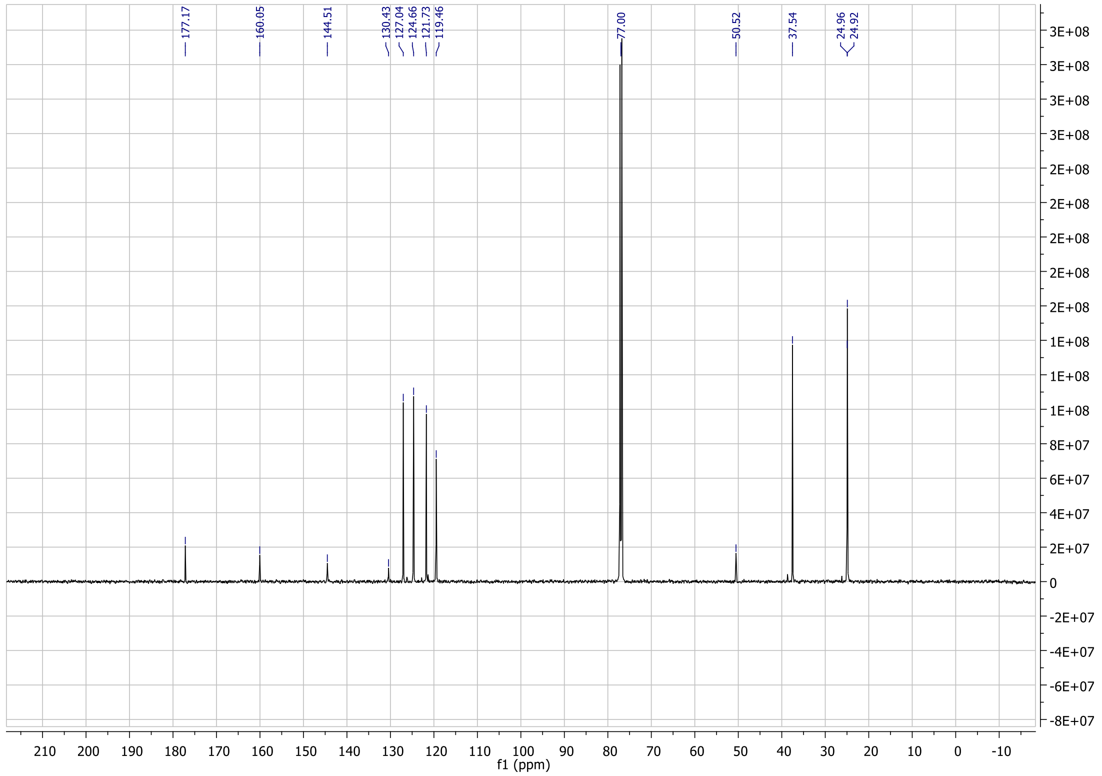

^{1}H NMR (600 MHz, $CDCl_3$) of compound **21**

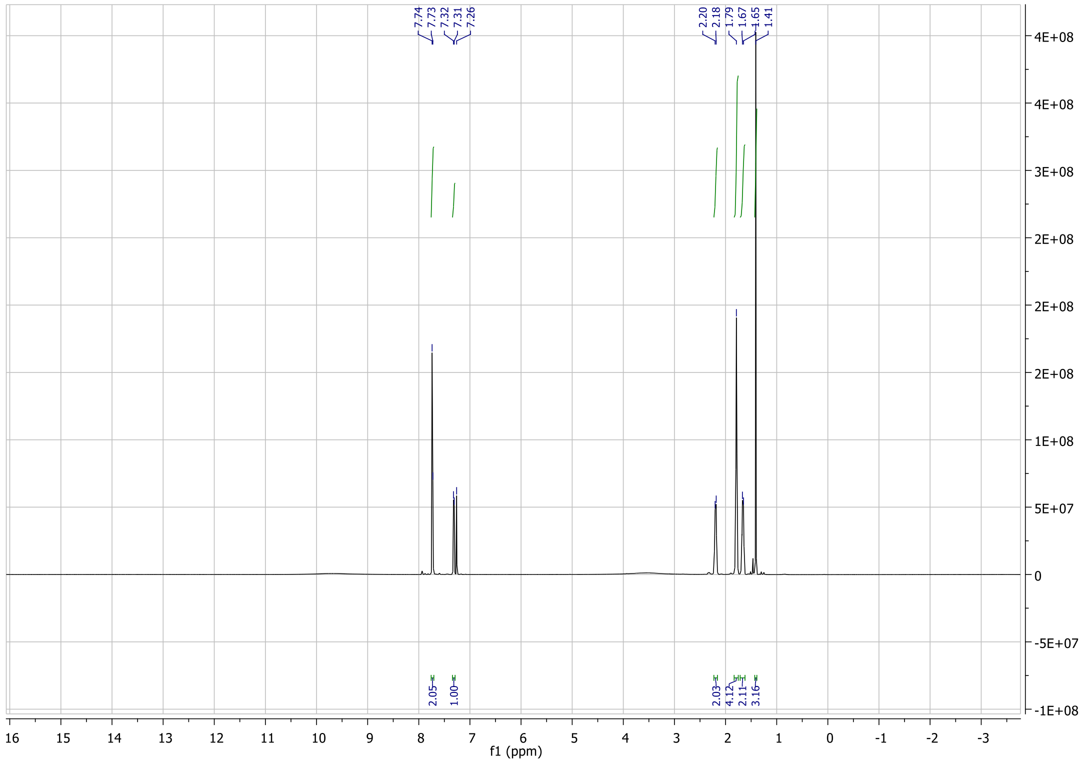

^{13}C NMR (150 MHz, $CDCl_3$) of compound **21**

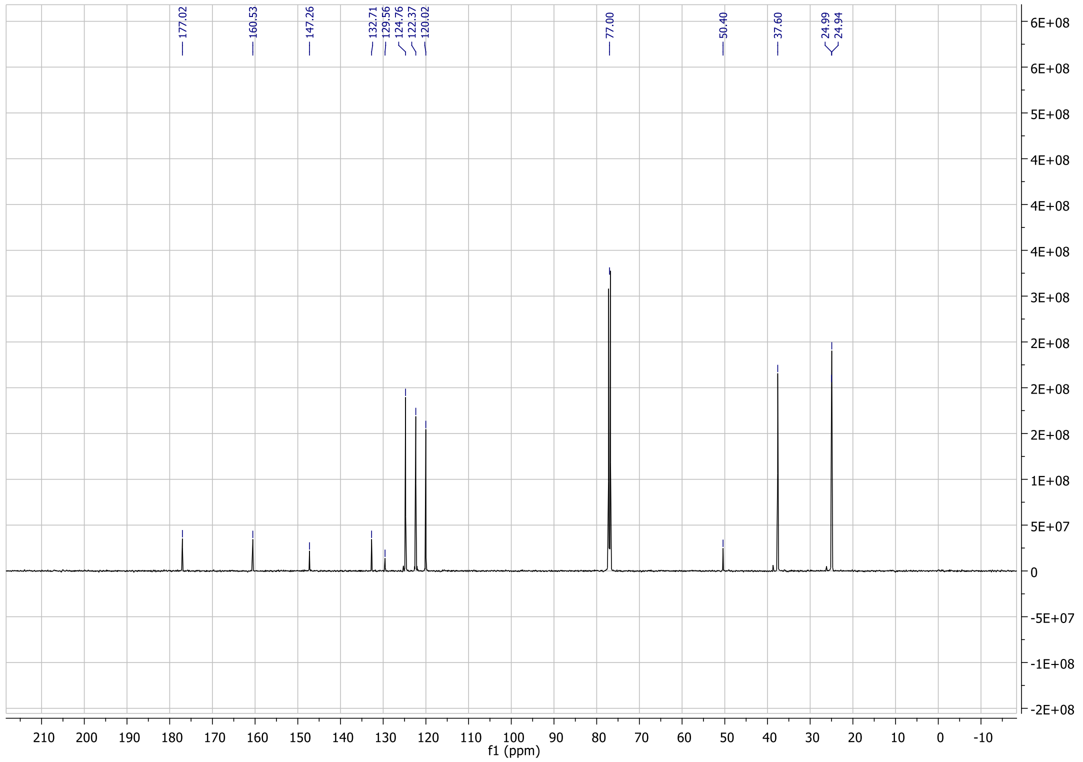

^{1}H NMR (600 MHz, $CDCl_3$) of compound **22**

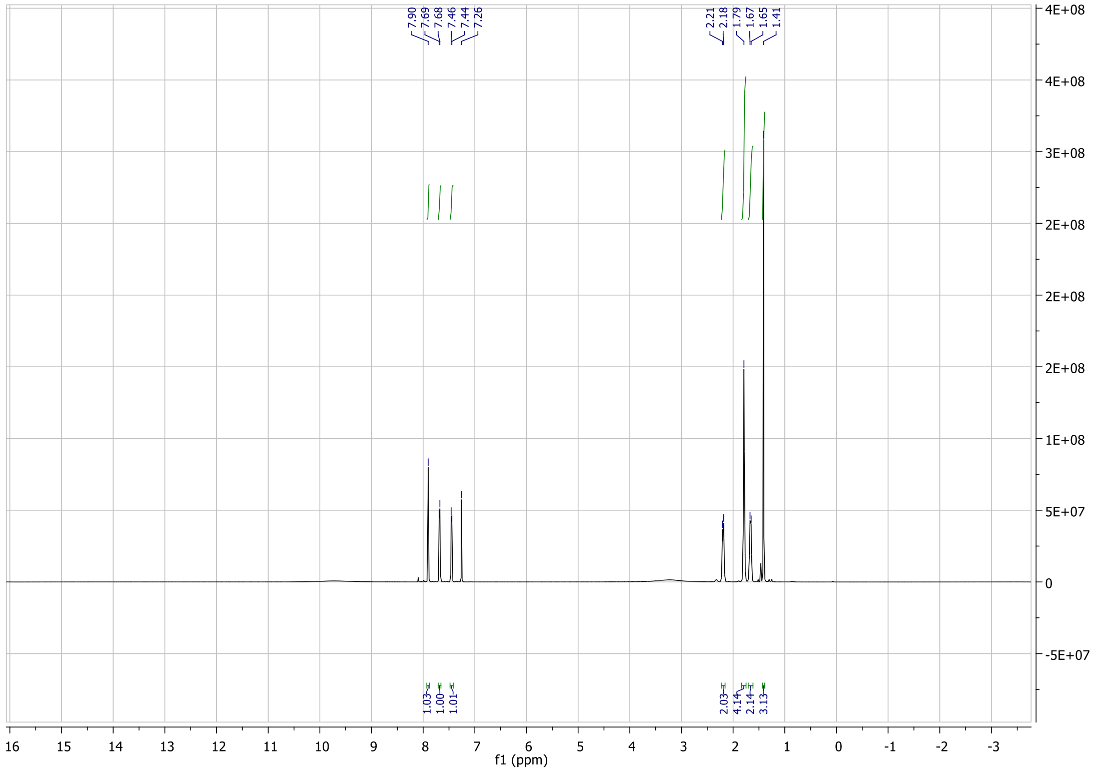

^{13}C NMR (150 MHz, $CDCl_3$) of compound **22**

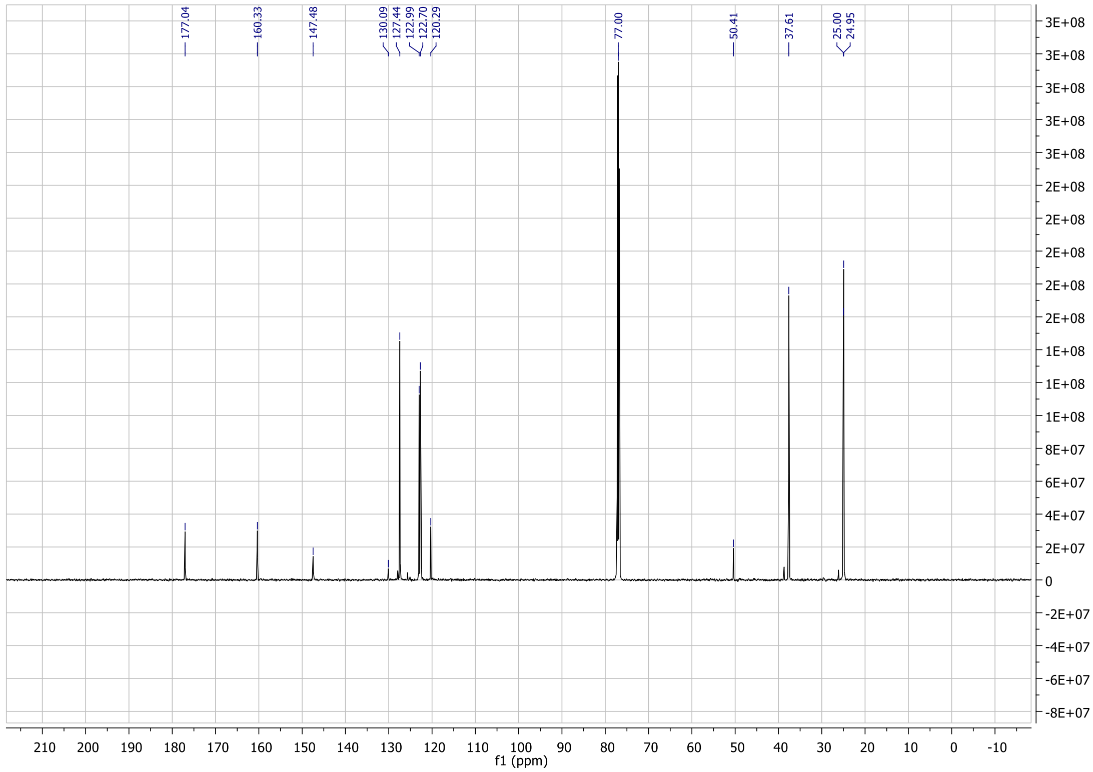

^{1}H NMR (600 MHz, $CDCl_3$) of compound **23**

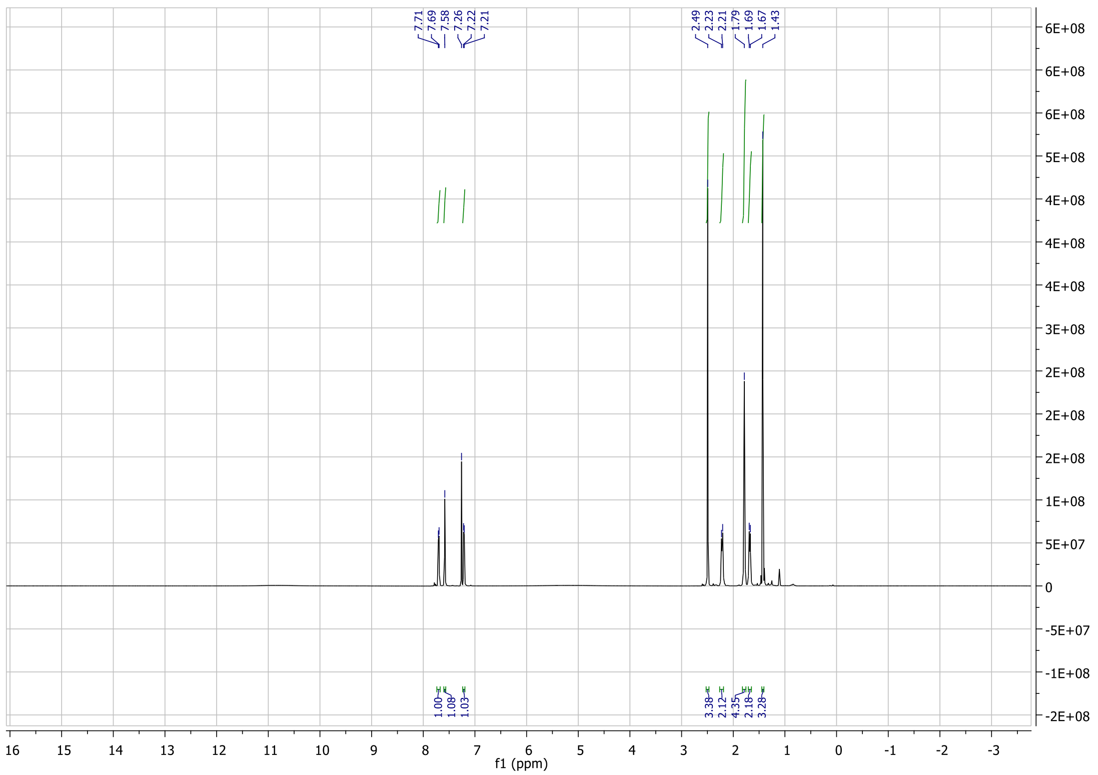

^{13}C NMR (150 MHz, $CDCl_3$) of compound **23**

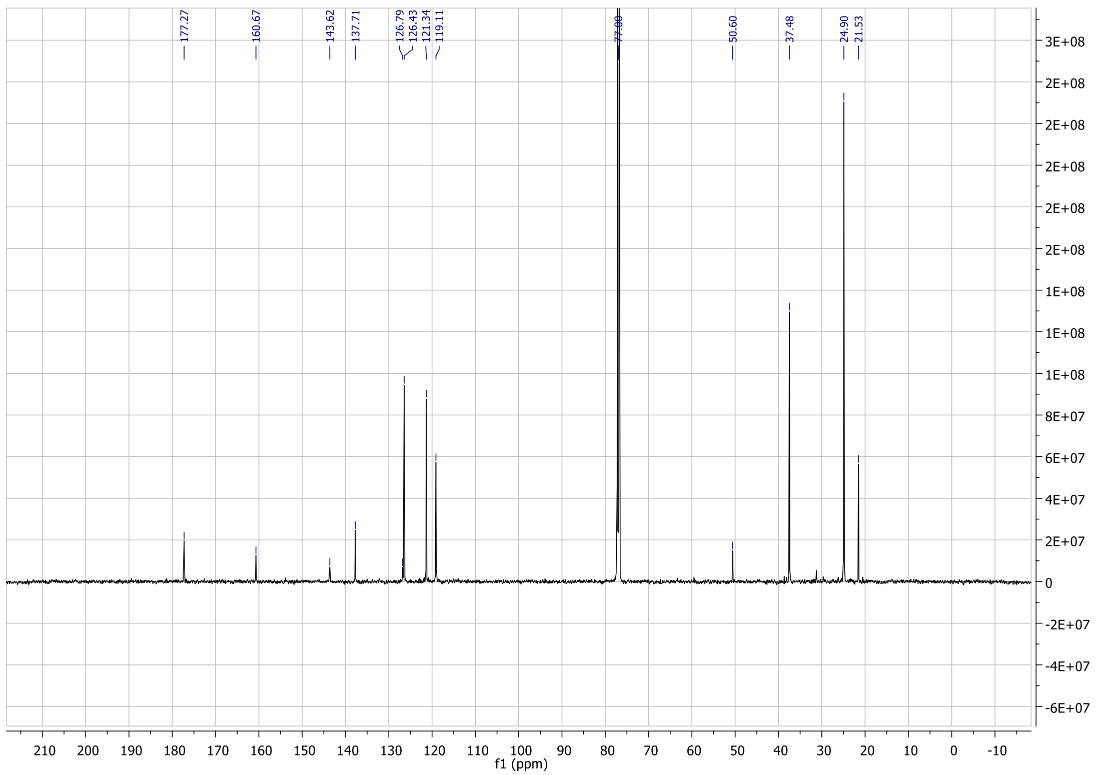

^{1}H NMR (600 MHz, $CDCl_3$) of compound **24**

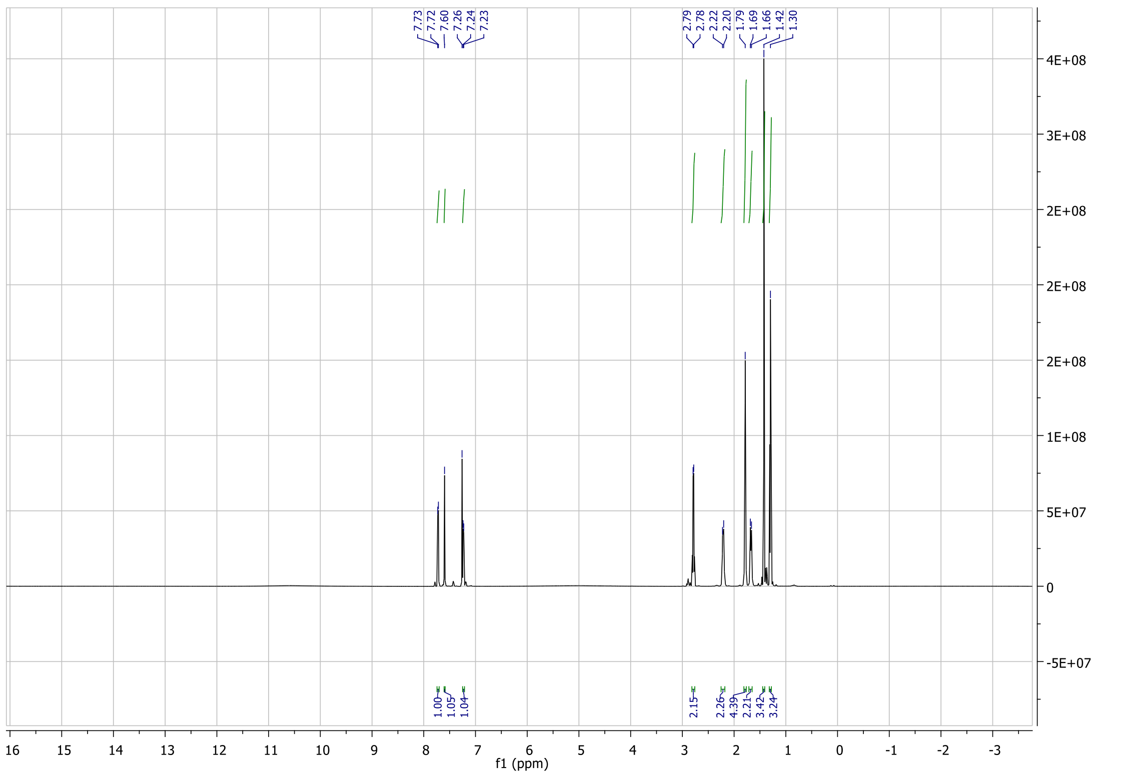

^{13}C NMR (150 MHz, $CDCl_3$) of compound **24**

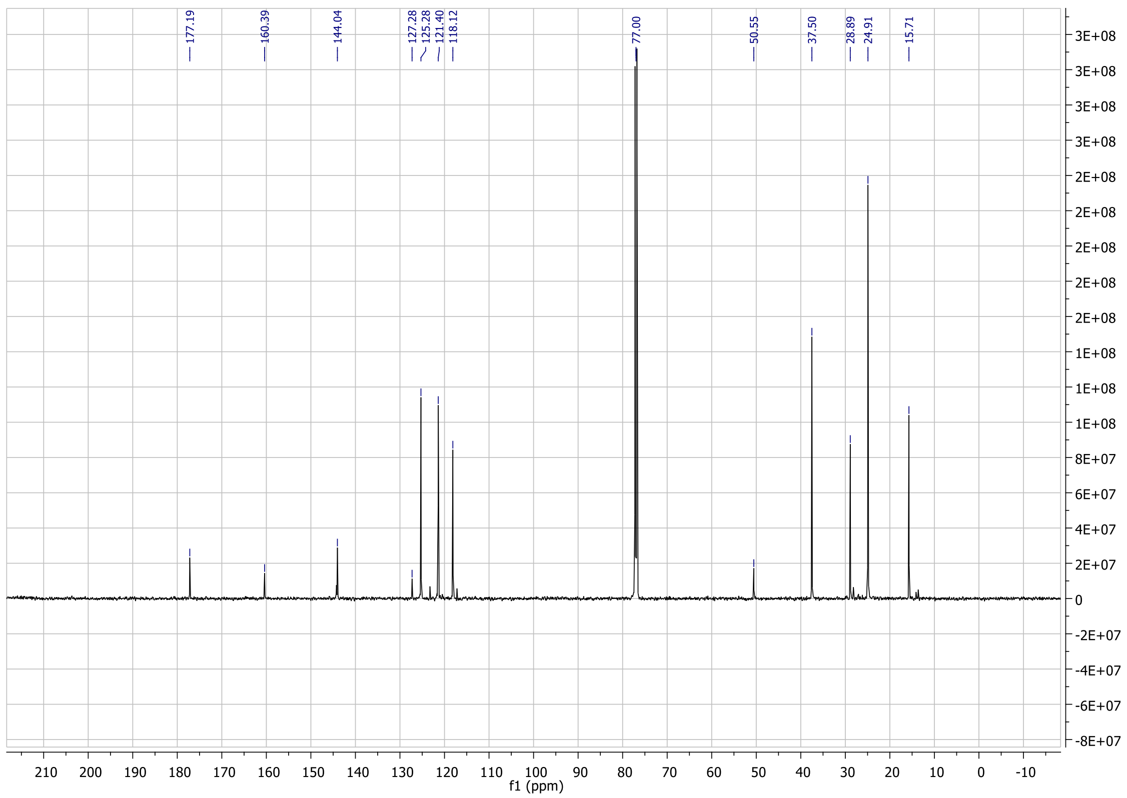

^{1}H NMR (600 MHz, $CDCl_3$) of compound **25**

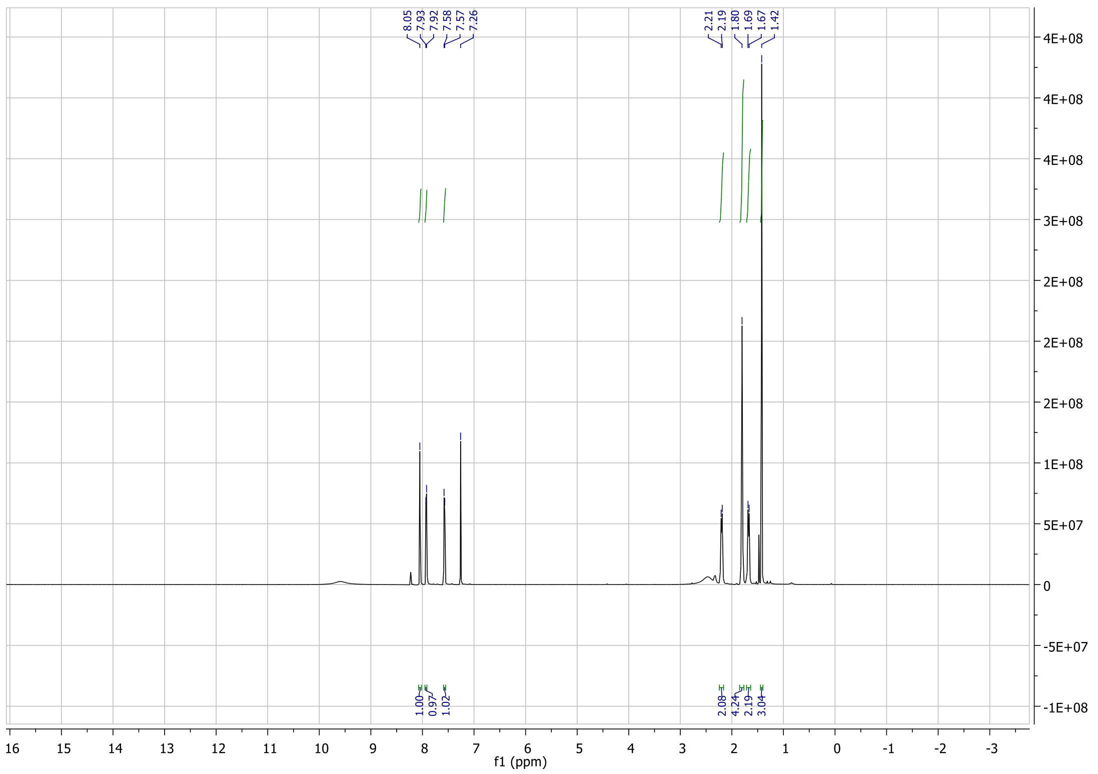

^{13}C NMR (150 MHz, $CDCl_3$) of compound **25**

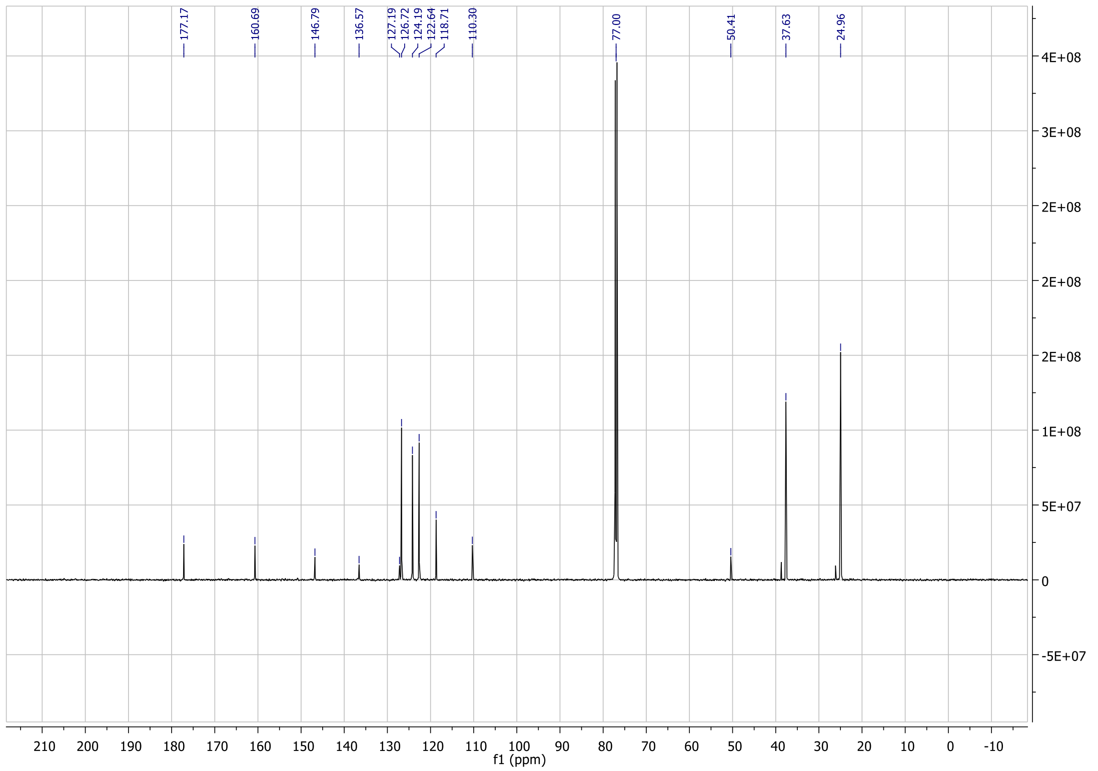

^{1}H NMR (400 MHz, DMSO-d6) of compound **26**

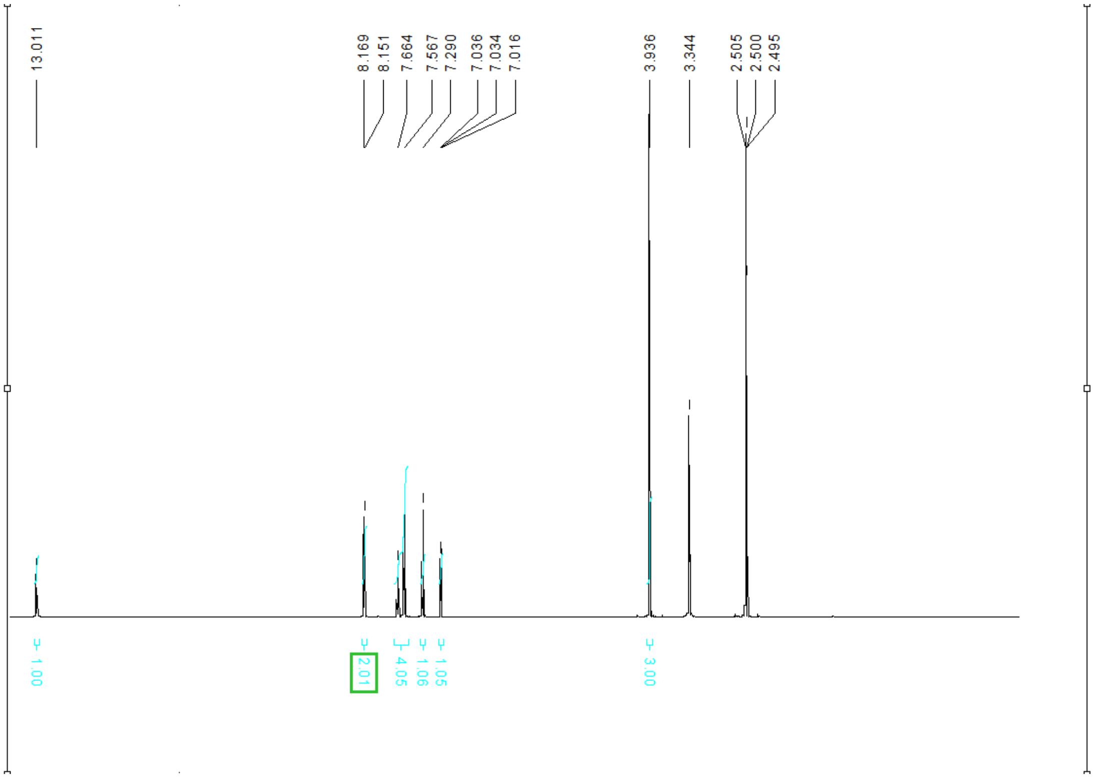

^{13}C NMR (100 MHz, DMSO-d6) of compound **26**

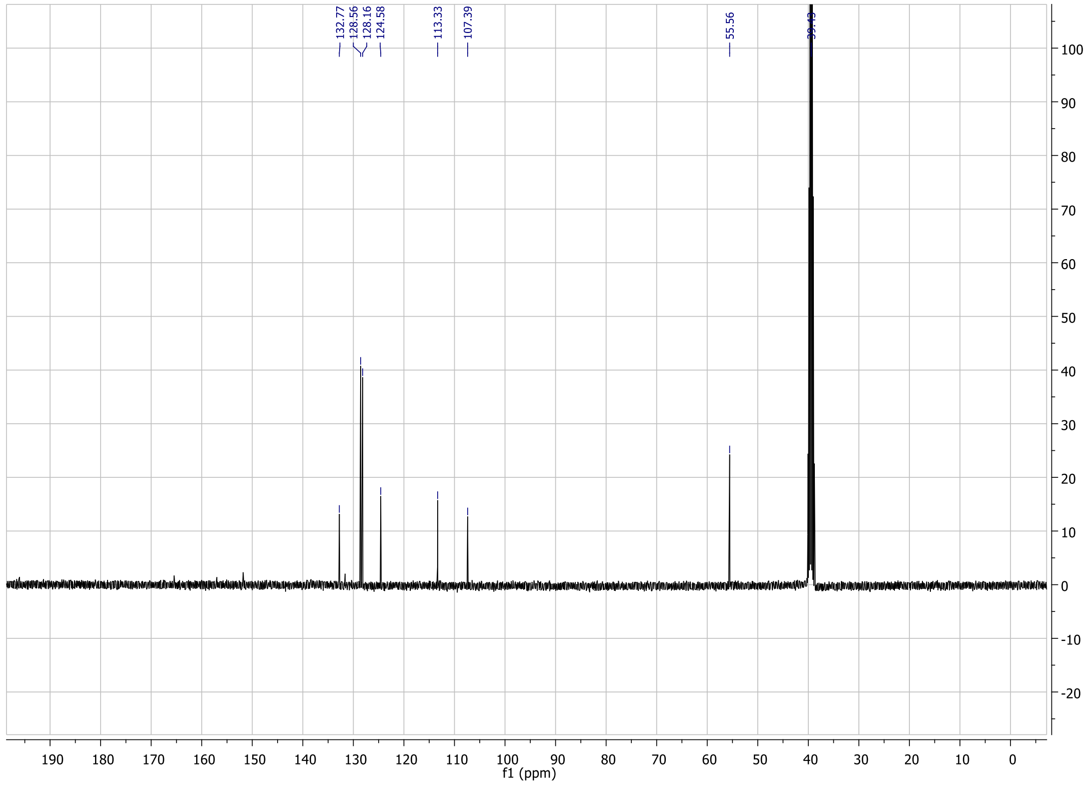

Supplementary References

1 R. K. Y. Cheng, E. Segala, N. Robertson, F. Deflorian, A. S. Doré, J. C. Errey, C. Fiez-Vandal, F. H. Marshall and R. M. Cooke, *Structure*, 2017, **25**, 1275-1285.e4.

2 P. Rucktooa, R. K. Y. Cheng, E. Segala, T. Geng, J. C. Errey, G. A. Brown, R. M. Cooke, F. H. Marshall and A. S. Doré, *Sci. Rep.*, 2018, **8**, 41.

3 D. Rodríguez, X. Bello and H. Gutiérrez-De-Terán, *Mol. Inform.*, 2012, **31**, 334–341.

4 H. J. C. Berendsen, D. van der Spoel and R. van Drunen, *Comput. Phys. Commun.*, 1995, **91**, 43–56.

5 W. L. Jorgensen, D. S. Maxwell and J. Tirado-Rives, *J. Am. Chem. Soc.*, 1996, **118**, 11225–11236.

6 O. Berger, O. Edholm and F. Jähnig, *Biophys. J.*, 1997, **72**, 2002–2013.

7 W. L. Jorgensen, J. Chandrasekhar, J. D. Madura, R. W. Impey and M. L. Klein, *J. Chem. Phys.*, 1983, **79**, 926–935.

8 P. Matricon, A. Ranganathan, E. Warnick, Z. G. Gao, A. Rudling, C. Lambertucci, G. Marucci, A. Ezzati, M. Jaiteh, D. Dal Ben, K. A. Jacobson and J. Carlsson, *Sci. Rep.*, 2017, **7**, 6398.

9 J. Marelius, K. Kolmodin, I. Feierberg and J. Åqvist, *J. Mol. Graph. Model.*, 1998, **16**, 213–225.

10 M. J. Robertson, J. Tirado-Rives and W. L. Jorgensen, *J. Chem. Theory Comput.*, 2015, **11**, 3499–3509.

11 G. King and A. Warshel, *J. Chem. Phys.*, 1989, **91**, 3647–3661.

12 J. P. Ryckaert, G. Ciccotti and H. J. C. Berendsen, *J. Comput. Phys.*, 1977, **23**, 327–341.

13 F. S. Lee and A. Warshel, *J. Chem. Phys.*, 1992, **97**, 3100–3107.

14 T. C. Beutler, A. E. Mark, R. C. van Schaik, P. R. Gerber and W. F. van Gunsteren, *Chem. Phys. Lett.*, 1994, **222**, 529–539.

15 R. W. Zwanzig, *J. Chem. Phys.*, 1954, **22**, 1420–1426.

16 J. B. Baell and G. A. Holloway, *J. Med. Chem.*, 2010, **53**, 2719–2740.
